# Novel anti-apoptotic L-DOPA precursors SuperDopa and SuperDopamide as potential neuroprotective agents for halting/delaying progression of Parkinson’s disease

**DOI:** 10.1038/s41419-022-04667-2

**Published:** 2022-03-11

**Authors:** Tom Wiesen, Daphne Atlas

**Affiliations:** Dept. of Biological Chemistry Institute of Life Sciences, The Hebrew University of Jerusalem, Jerusalem, 91904 Israel

**Keywords:** Chemical modification, Parkinson's disease

## Abstract

Parkinson’s disease (PD) is characterized by a gradual degeneration of the dopaminergic neurons in the substantia nigra pars compacta (SNpC). Levodopa, the standard PD treatment, provides the missing dopamine in SNpC, but ultimately after a honeymoon with levodopa treatment the neurodegenerative process and the progression of the disease continue. Aimed at prolonging the life of dopaminergic cells, we prepared the levodopa precursors SuperDopa (SD) and SueprDopamide (SDA), in which levodopa is merged with the antioxidant N-acetylcysteine (NAC) into a single molecule. Rotenone is a mitochondrial complex inhibitor often used as experimental model of PD. In vivo, SD and SDA treatment show a significant relief of motor disabilities in rotenone-injected rats. SD and SDA also lower rotenone-induced-α-synuclein (α-syn) expression in human SH-SY5Y cells, and α-syn oligomerization in α-syn-overexpressing-HEK293 cells. In the neuronal SH-SY5Y cells, SD and SDA reverse oxidative stress-induced phosphorylation of cJun-N-terminal kinase (JNK) and p38-mitogen-activated kinase (p38^MAPK^). Attenuation of the MAPK-inflammatory/apoptotic pathway in SH-SY5Y cells concurrent with protection of rotenone-triggered motor impairment in rats, is a manifestation of the combined antioxidant/anti-inflammatory activity of SD and SDA together with levodopa release. The concept of joined therapies into a single molecule, where levodopa precursors confer antioxidant activity by enabling NAC delivery across the BBB, provides a potential disease-modifying treatment for slowing PD progression.

## Background

Neurodegenerative diseases share neuroinflammation as a common mechanism resulting from activated microglia that release pro-inflammatory cytokines. The loss of 9-type dopaminergic (DA) neurons in the substantia nigra pars compacta (SNpc) has been shown to be responsible for the first motor symptoms of Parkinson’s disease (PD).

Virtually all PD patients will require levodopa therapy. However, PD progresses with time, and patients experience diminished duration of benefit from each dose. The diminished effectiveness over time is also accompanied by the development of medication-related complications such as motor fluctuations, and levodopa-induced dyskinesia [1]. The decrease in efficacy of levodopa is attributed mainly to the continuous loss of DA cells, presenting an unmet medical need with no approved drug therapy. It requires a disease-modifying approaches that would delay or slow the clinical progression of the disease (reviews [1–3]).

Although the cause of neurodegeneration processes in sporadic PD is not fully understood, constitutive production of reactive oxygen species (ROS) during the oxidative metabolism of dopamine within the DA cells, and impaired bioenergetics in the mitochondria contribute to premature cell death. Oxidative stress also triggers the mitogen-activated protein kinases (MAPKs) inflammatory/apoptotic pathways through the phosphorylation of cJun N-terminal kinase (JNK) and p38-mitogen protein kinase (p38^MAPK^) (review [4–7]).

Mitochondrial toxins including herbicide paraquat, pesticide rotenone, or the funcocide MB-manganese, target a variety of redox-sensitive proteins in the brain contributing to sporadic PD [8]. These toxins induce mitochondrial electron-transport chain dysfunction, mainly through complex1 inhibition, and are often used as disease models for PD. Similar to these environmental mitochondrial toxins, PD-linked gene mutations have been shown to be associated with mitochondrial damage combined with oxidative/nitrosative stress [9]. An increase in the basal and mitochondrial toxin-induced nitrosative stress resulted in inhibition of transcriptional activity of a critical cysteine (Cys) residue in myocyte-specific enhancer factor 2C, which is a redox-mediated protein that enhances DA neurons in alpha-synuclein mutant (α-syn^A53T^) A9 DA neurons [10].

Oxidative/nitrosative stress induced by paraquat has been shown to modify α-syn by nitration of a tyrosine residue and oxidation of a methionine residue, thereby contributing to its aggregation [11–13]. These results are consistent with the identification of nitrosative/oxidative stress as one of the players in α-syn aggregation, and provide a molecular link to the cascade of events leading to the selective death of SNpc DA neurons in PD. The accumulation of α-syn may also impair mitochondrial homeostasis by decreasing the activity of mitochondrial complex1 [14]. The wt α-syn and the mutated α-syn^A53T^ are linked to genetic mutations that generate intracellular inclusions called Lewy body that contribute to the neuronal dysfunction and pathology of DA cells [15–17]. These aberrant protein aggregations are one of PD hallmark and are associated with death of dopamine-producing cells [18–20].

To develop a disease-modifying strategy for PD, we designed two dopamine precursors, AcCys-L-Dopa-Cys-amide, called SuperDopa (SD), and AcCys-L-Dopa amide, called SuperDopAmide (SDA).

SD is a family member of the thioredoxin mimetic peptides (TXM-peptides). TXM- peptides protect neuronal and non-neuronal cells from apoptosis in vitro and in vivo, by inhibiting the oxidative stress-induced MAPK-inflammatory/apoptotic pathway and catalyzing S-denitrosylation [7, 21–30].

SDA is a dipeptide comprising of N-acetylcysteine (NAC) and DopAmide. DopAmide itself is a levodopa precursor that confers a sustained release of dopamine in 6-OH-dopamine-lesioned rats [31].

To recapitulate in vivo features of SD and SDA we used the highly reproducible rotenone rat model, in which rotenone-treated rats develop PD features like bradykinesia, postural instability and/or rigidity, thus providing an excellent tool to test potential PD neuroprotective reagents [32]. In vitro, SD and SDA effects on the anti-apoptotic/anti-inflammatory activity and on α-syn aggregation were investigated in human neuroblastoma SH-SY5Y cells and in HEK293 overexpressing α-syn.

As demonstrated, both SD and SDA appeared to combine anti-inflammatory/antioxidant activities with the ability to replenish the cells with levodopa. The impact of joined activities into a single molecule is often greater than administrating each molecule separately.

The concept of combined therapies of levodopa precursor with antioxidant activity into a single molecule that enables NAC delivery across the BBB and inhibition of the neurodegenerative process, could become a disease-modifying treatment with an outlook for slowing PD progression.

## Results

### Design and synthesis of SuperDopa (SD)

**Fig. 1:**
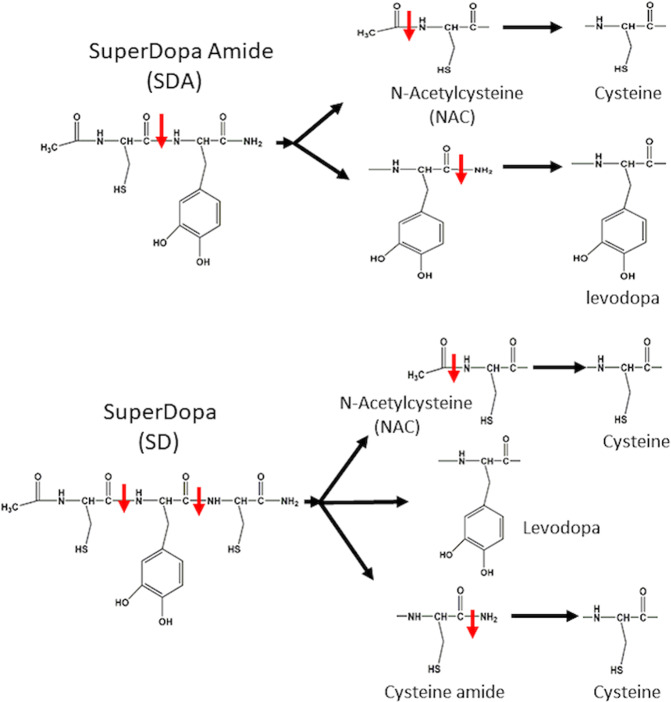
The structure and putative proteolysis sites of SuperDopa (SD) and SuperDopaAmide (SDA). Upper, putative proteolysis sited for SuperDopa (SD) and lower, putative proteolysis sited for SuperDopaAmide (SDA). The red arrows indicate peptide bond, potential cleavage sites at the peptides bonds.

SD (AcCys-L-DOPA-Cys-amide) is N-acetylated tri-peptide comprising of two Cys residues that flank levodopa (Fig. 1; upper). SD was synthesized (Novetide, Ltd) and its purity (98%) and molecular weight were determined by HPLC (Fig. S1), and mass spectra (Fig. S2). The acetylation at the amino-terminal and the α-C-amidation of SD, neutralize the positive and negative charges of the peptide. These modifications increase lipophilicity and lead to enhanced membrane targeting and cell membrane permeation. SD is structurally analogous to the TXM-peptide, TXM-CB3 (AcCys-Pro-Cys-amide) that mimics thioredoxin activity [21–23, 26–28]. As a peptide, SD is susceptible to cleavage by endo-and amino-peptidases (Fig. 1; upper; red arrows). The intracellular proteolysis of SD generates levodopa, NAC, and Cys-amide. NAC and Cys-amide are hydrolyzed further, to yield two Cys residues. Both NAC and Cys are reducing regents known as glutathione (GSH) precursors. In addition, cleavage of SD releases levodopa. The non-charged SD, similar to TXM-CB3, is predicted to cross the BBB [24], enabling transport of levodopa, NAC, and Cys across the BBB onto DA neurons.

### Design and synthesis of SuperDopAmide (SDA)

SDA (N-Acetylcysteine-L-DOPA-amide) is an N-acetyl blocked dipeptide comprising of a single Cys and a single residue of levodopa modified at the carboxy-terminal to an amide. SDA was synthesized (Novetide, Ltd), and its purity (98%) and molecular weight were determined by HPLC (Fig. S3), and mass spectra (Fig. S4). SDA proteolysis (Fig. 1; lower; red arrow) generates NAC, and levodopa-amide (DopAmide) (Fig. 1; lower, red arrow). DopAmide itself is a levodopa precursor, which is hydrolyzed to levodopa and replenishes cellular dopamine [31]. The multiple activities of SDA, NAC and elevating levodopa, are predicted to provide neuroprotection simultaneously with increasing dopamine levels.

### In vivo studies

Here, SD and SDA were tested in vivo using the rotenone rat model, which mimics PD motor dysregulations and is one of the most reliable animal models of PD. Three groups of rats were injected intraperitoneally (i.p) in the morning with rotenone (3.0 mg/kg/day) in a specialized vehicle, for 9 days. In the afternoon, one group was i.p injected with SDA (33 mg/kg/day) and another group, with SD (33 mg/kg/day), for 9 days (“Methods”).

**Fig. 2:**
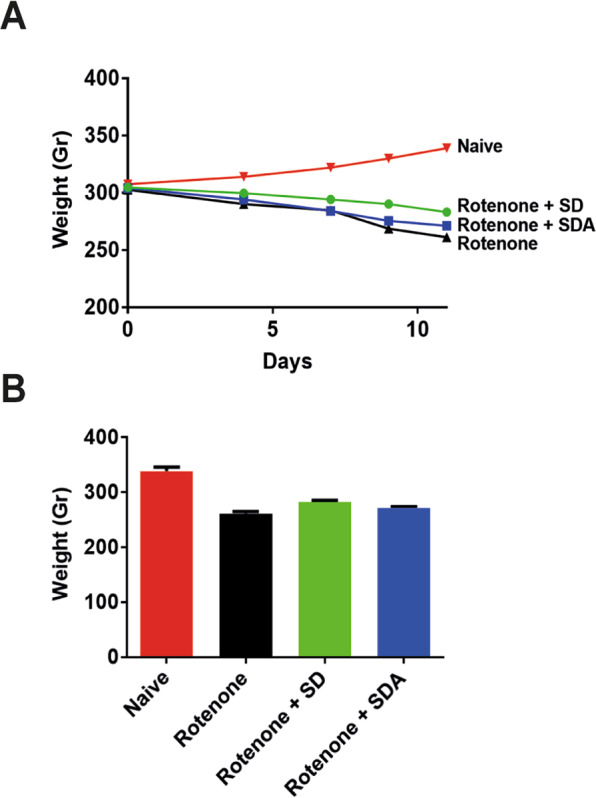
The effect of rotenone, rotenone + SD, and rotenone + SDA on body mass. **A** Daily intraperitoneal rotenone (3 mg/kg) for 9 days elicits moderate change in body mass from day 0 to day 10 in rats (300 g; *n* = 6). **B** Quantification of body mass taken at day 0, 4, 7, and 10 after rotenone injection.

Body weight was monitored on days 4, 7, and 10 (Fig. 2A). A slight reduction in body weight in rotenone/only injected rats was observed, compared to naive rats, and no significant decrease was observed in rats treated with rotenone/SD or rotenone/SDA, as quantified on day 10 (Fig. 2B). The rotenone/only-treated rats developed bradykinesia, postural instability, and/or rigidity, which were not observed in the rotenone/SD and rotenone/SDA-treated rats.

#### SD and SDA prevent motor impairment in the rotenone rat model

##### Rotarod behavior test

**Fig. 3:**
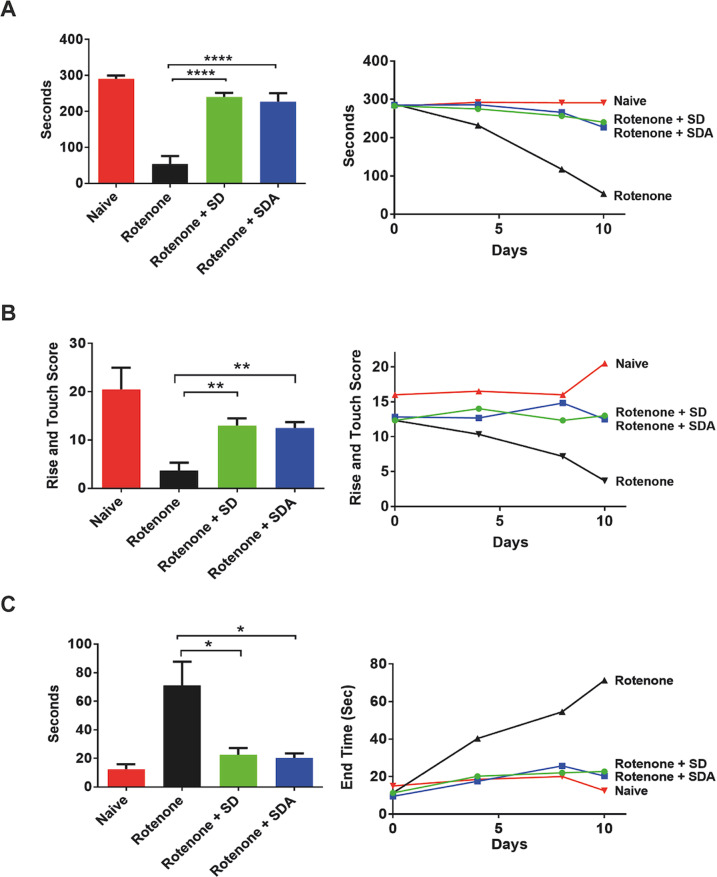
SD and SDA rescue motor impairment in rotenone-treated rats. Rats were injected with rotenone alone (3 mg/kg; 10 days), or with either SD (33.3 mg/kg daily for 9 days) or SDA (33.3 mg/kg daily; 9 days). Motor activity was examined at days 4, 7, and 10 by **A** Rotarod assay monitored motor coordination. Apparatus was set to accelerate from 4 to 40 rpm in 300 s, and animals placed in separate lanes riding time **B** the Cylinder test which evaluated locomotor asymmetry by placing the rats in a clear glass cylinder (40 cm high and 20 cm diameter) and number of rears were counted during 5 min and (**C**) the walk bean assay which measured successful walk rats walk across an elevated 1 m long aluminum beam, counting the number of foot slips from the beam as a measure of locomotor co-ordination and grip-strength.

Motor coordination and balance were evaluated using the rotarod behavior test at days 4, 7, and 10 (Fig. 3A left). The behavioral performance of treated rats compared to naive rats was quantified on day 10 (Fig. 3A, right). A statistically significant difference in performance was observed between rotenone-treated rats and naive rats. In sharp contrast, rats treated with rotenone together with SDA or SD, showed longer timing on the rotarod, indicating a significant protection of motor activity, compared to rotenone only/treated rats. Performance on rotarod of rotenone/SD or rotenone/SDA treated rats was similar to naive rats.

##### Rat-rearing behavior test (*Cylinder assay*)

The rearing cylinder test was used to evaluate locomotor activity in rodent models of CNS disorders. A gradual decrease in the ability of the rotenone-treated rats to place the palm on the wall of the cylinder was monitored at days 4, 7, and 10, indicating impaired body support (Fig. 3B, left). Rats treated with rotenone/SD or rotenone/SD displayed rearing activity similar to naive rats, showing a significant improvement of rearing activity. The performance of rearing activity was quantified on day 10 (Fig. 3B, right).

*Rat beam walk test* was applied for examining SDA and SD effects on motor coordination and balance in the rotenone rat model. The goal of this assay was to test the rat ability to stay upright and walk across an elevated narrow beam to a safe platform. After 2 days training, the assay examined the time it takes for the rat to traverse the beam and the number of paw slips that occur in the process. This test is complementary to the rotarod, and can detect subtle deficits in motor skills and balance.

A significant improvement in the time required for terminating the walk was observed in the rotenone/SD or rotenone/SDA treated rats, compared to rotenone/only, at days 4, 7, and 10 (Fig. 3C left). Quantified at day 10, the rotenone/only-treated rats displayed a significant longer time to complete the beam walk compared to rotenone/SDA or rotenone/SD treated rats, (Fig. 3C, right). The central nervous system-mediated locomotor activity, which was protected subsequent to i.p injection of SD and SDA, indicates BBB permeation of both compounds.

### In vitro studies

To assess a putative neuroprotective mechanism, and cell permeation we examined the antioxidant/anti-inflammatory activities of SD and SDA, by monitoring the oxidative stress-induced MAPK-apoptotic/inflammatory pathway in human neuroblastoma SH-SY5Y cells. Oxidative stress was induced by auranofin (Auf), an inhibitor of thioredoxin reductase. SH-SY5Y cells are often used as a cellular model of various neurological oxidative stress-related disorders such as PD and Alzheimer’s disease [33, 34].

### Mitochondrial membrane potential

Initially, we explored the effects of Auf on the mitochondria, recording mitochondrial membrane potential (MMP). Similar to rotenone and paraquat, we found that Auf lowered the MMP (Fig. S5). These results also confirm previous studies showing mitochondrial dysfunction by Auf [35].

### SD, SDA, and TXM-CB3 reverse the Auf-induced morphological changes in SH-SY5Y cells

Next, we wanted to assess the effects of SD and SDA on Auf- or rotenone-induced cell morphology. SH-SY5Y were incubated with 3 µM Auf for 30 min, washed, and incubated for 3.5 h at 37 °C with or without SD or SDA. As shown in Fig. S6, morphological changes, appeared 4 h after Auf-treatment, and were accompanied with a reduction in cell number, and a loss of cell-to-cell contact. These changes were reversed in cells incubated with either SD, SDA, or TXM-CB3 (Fig. S6). No change however, was observed in cell morphology in cells incubated with 5 µM rotenone for 3.5 h at 37 °C (Fig. S7). Previous studies quantified the Auf effects on cell viability and early and late apoptosis [23].

### SD and SDA reverse Auf-induced JNK phosphorylation in SH-SY5Y cells

**Fig. 4:**
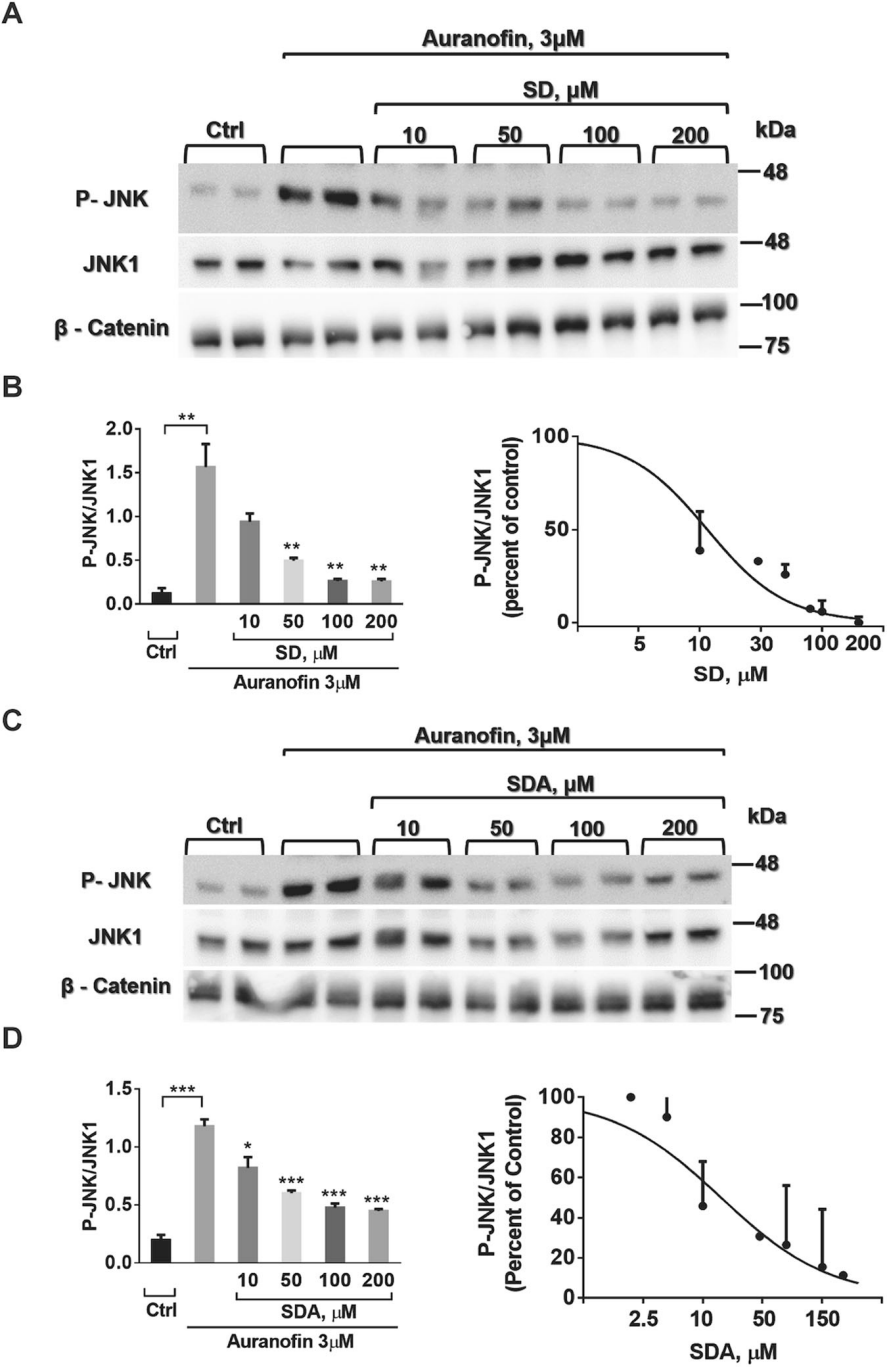
SD and SDA reverses the auranofin-induced phosphorylation of JNK in human neuroblastoma SH-SH5Y cells. **A**, **B** SH-SY5Y cells were incubated with 3 μM auranofin (Auf) for 30 min, washed, and treated with or without increasing concentrations of SD in 4 independent experiments. **C**, **D** SH-SY5Y cells were incubated with 3 μM auranofin (Auf) for 30 min, washed, and treated with or without increasing concentrations of SDA in 3 independent experiments, as indicated. Cell lysates proteins in equal amounts were separated on 10% SDS-PAGE, and analyzed by immunoblotting with the corresponding antibodies. The blots were cut prior to hybridization with antibodies shown in Fig. S8. The ratios of phosphorylated JNK to unphosphorylated JNK, or β-catenin were calculated. The values shown are averages (±SEM) based on four independent experiments, normalized to the phosphorylation state of cells treated with Auf after 3.5 h and plotted with a linear regression program. Student’s *t*-test (two populations) was performed for AuF treated cells. *P value < 0.05; **P value < 0.01; ***P value < 0.005.

Next, to investigate the possible anti-apoptotic/anti-inflammatory activity of SD and SDA we explored Auf-induced JNK phosphorylation in SH-SY5Y cells. The cells were treated with 3 μM Auf for 30 min, washed, and then incubated for 3.5 h at 37 °C with or without SD (Fig. 4A) or SDA (Fig. 4B) (see Fig. S8), at the indicated concentrations. JNK1/2 phosphorylation was monitored by western blot analysis using the anti-phospho JNK1 antibodies, and normalized to total JNK1 or β-catenin, with the corresponding anti-JNK1 or anti-β-catenin antibodies. The reduction in Auf-induced JNK phosphorylation mediated by SD or SDA was concentration-dependent with apparent dissociation constant of Ki = 50.2 ± 1.1 µM, and Ki = 14.2 ± 1.4 µM, respectively. Hence, by inhibiting JNK1 phosphorylation, SD and SDA inhibit the MAPK-inflammatory/apoptotic pathway.

### SD and SDA reverse Auf-induced p38^MAPK^ phosphorylation

**Fig. 5:**
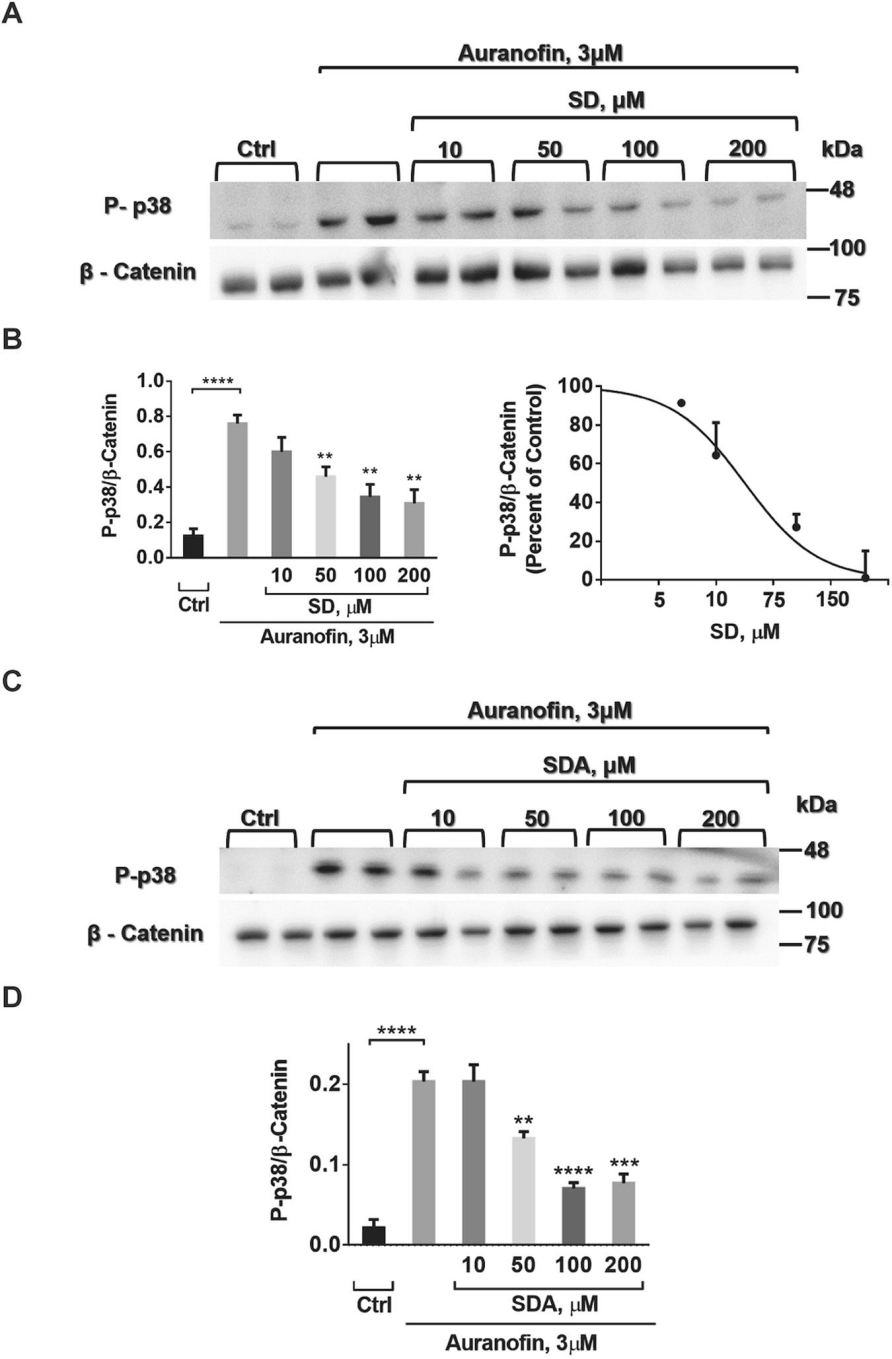
SD and SDA reverse Auf-induced p38^MAPK^ phosphorylation in human neuroblastoma SH-SY5Y cells. **A**, **B** SH-SY5Y cells were incubated with 3 μM Auf for 30 min, washed, and treated with or without increasing concentrations of SD. **C**, **D** SH-SY5Y cells were incubated with 3 μM Auf for 30 min, washed, and treated with or without increasing concentrations of SDA for 3.5 h. Proteins in equal amounts of cell lysates were separated on 10% SDS-PAGE, and analyzed by immunoblotting with the corresponding antibodies. The blots were cut prior to hybridization with antibodies shown in Fig. S9. Phosphorylation of p38^MAPK^ was quantified by immunoblot densitometry. The ratios of phosphorylated p38^MAPK^ to the housekeeping β-catenin were calculated based on three independent experiments. The values shown are averages (±SEM) normalized to the phosphorylation state of cells treated with Auf after 3.5 h and plotted with a linear regression program. Student’s *t*-test (two populations) was performed for Auf treated cells. *P value < 0.05; **P value < 0.01; ***P value < 0.005.

Next, the ability of SD and SDA to reverse Auf-induced p38^MAPK^ phosphorylation was examined in SH-SY5Y cells. The cells were treated for 30 min with 3 μM Auf, washed, and incubated for 3.5 h at 37 °C, with or without SD (Fig. 5A) or SDA (Fig. 5B) (see Fig. S9), at the indicated concentrations. Phosphorylation of p38^MAPK^ was monitored by western blot analysis using anti-phospho-p38^MAPK^ antibodies and normalized to β-catenin using anti β-catenin antibodies. The reduction in Auf-induced p38^MAPK^ phosphorylation mediated by SD and SDA was concentration-dependent, displaying an apparent dissociation constant Ki = 17.9 ± 1.2 µM, and Ki = 11.2 ± 1.4 µM, respectively.

Hence, SD and SDA appear to reverse Auf-induced apoptosis through the ASK1-MEK/JNK or ASK1-MEK/p38^MAPK^ pathways. Similar anti-apoptotic activity of a variety of other TXM-peptides was previously reported [21, 23, 24].

### The effect of SD on Auf-induced ERK1/2 phosphorylation

As opposed to JNK and p38^MAPK^, SD showed no significant decrease in Auf-triggered phosphorylation of the Extracellular signal Regulated Kinases (ERK1/2) (Fig. S10). This result is consistent with a marginal reversal of the Auf-induced ERK1/2 phosphorylation, previously reported for the TXM-peptide TXM-CB3 [23, 24].

### Levodopa ethyl ester (LDEE) does not reverse oxidative stress-induced activation of JNK or p38 apoptotic pathway

The antioxidant activity of SD and SDA was compared to another levodopa precursor, LDEE [36], testing the reversal of Auf-induced JNK, and p38^MAPK^ phosphorylation in SH-SY5Y cells. The cells were treated for 30 min with 3 μM Auf, washed, and then incubated for 3.5 h at 37 °C, with or without increasing concentrations of LDEE (Fig. S11). As opposed to SD and SDA, LDEE did not reverse the Auf-induced phosphorylation of JNK or p38^MAPK^ at concentrations up to 200 µM (Fig. S11**)**.

**Fig. 6:**
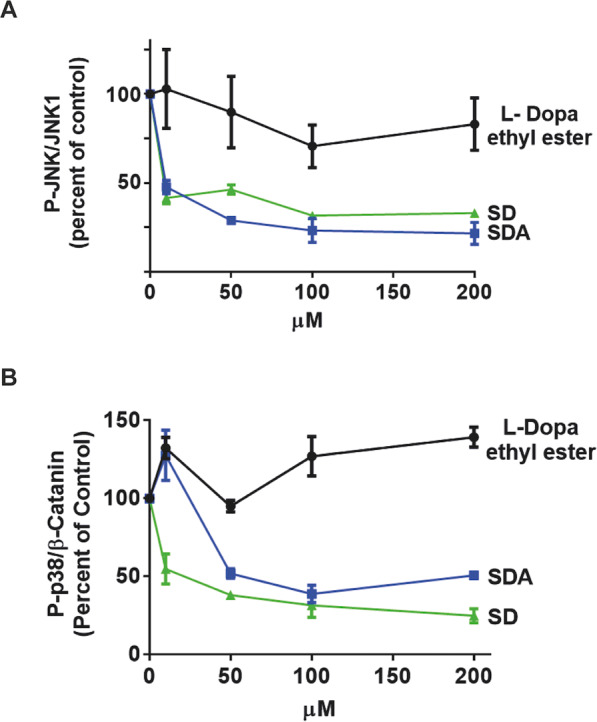
SD, SDA but not LDEE, reverse Auf-induced JNK and p38^MAPK^ phosphorylation. A concentration-dependent reduction in Auf-induced phosphorylation of **A** JNK and **B** p38^MAPK^ by SD, SDA, and LDEE (see legend and blots in Fig. S11). The ratios of phospho-JNK/JNK1 and phospho-p38^MAPK^/β-catenin were calculated as percentage of control based on two independent experiments (±SEM) for each of the compounds.

The effects of the three precursors SD, SDA, and LDEE, on Auf-induced JNK and p38^MAPK^ phosphorylation are compared (Fig. 6). SD and SDA but not LDEE, exhibited a concentration-dependent decrease in Auf-induced phosphorylation of JNK and p38^MAPK^ (Fig. 6A, B). These findings show the antioxidant activity of SD and SDA, which could be attributed to the thiol group of the Cys residue, most likely through preventing the dissociation of Trx/ASK1 [23].

### The effects of SD and SDA on rotenone-induced expression of α-synuclein (α-syn)

**Fig. 7:**
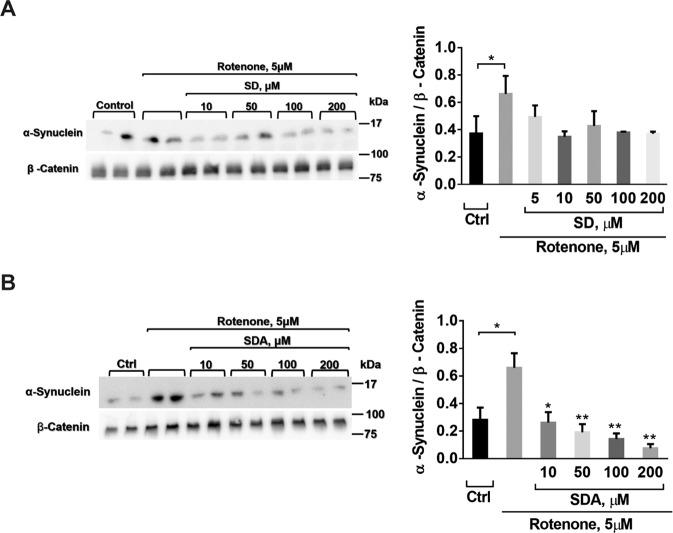
SD and SDA lower rotenone-induced α-synuclein level in SH-SY5Y cells. SH-SY5Y cells were treated with **A** SD or **B** SDA at the concentrations as indicated, for 60 min. Then the cells were washed and incubated with 5 µM rotenone for 16 h. Equal amounts of proteins of cell lysates were loaded and separated on 12% SDS-PAGE. The level of α-syn in the cell lysates was determined using α-synuclein antibodies. The blots were cut prior to hybridization with the corresponding antibodies, shown in Fig. S12. The values calculated by densitometry shown (*right*) are averages (±SEM) of two independent experiments normalized with housekeeping level of β-catenin; Student’s t-test (two populations) was performed for rotenone treated cells. *P value < 0.05; **P value < 0.01.

Copper ions play a role in synucleopaties, while the Cys-sulfhydryl group are known as copper chelators. These activities led us to investigate the effect of SD or SDA on rotenone-induced expression of α-syn in SH-SY5Y cells. Cells treated for 60 min with SDA (Fig. 7A) or SD (Fig. 7B) at the indicated concentrations, were washed, and then incubated with 5 µM rotenone for 24 h. The expression of α-syn was determined by western blot analysis, using α-syn antibodies normalized to β-catenin. Incubation of the cells with rotenone-triggered α-syn expression, was significantly higher than in untreated cells, and markedly suppressed in 10 µM of SD or 10 µM SDA-treated cells (see Fig. S12**)**

### SDA lowers α-synuclein (α-syn) aggregation

**Fig. 8:**
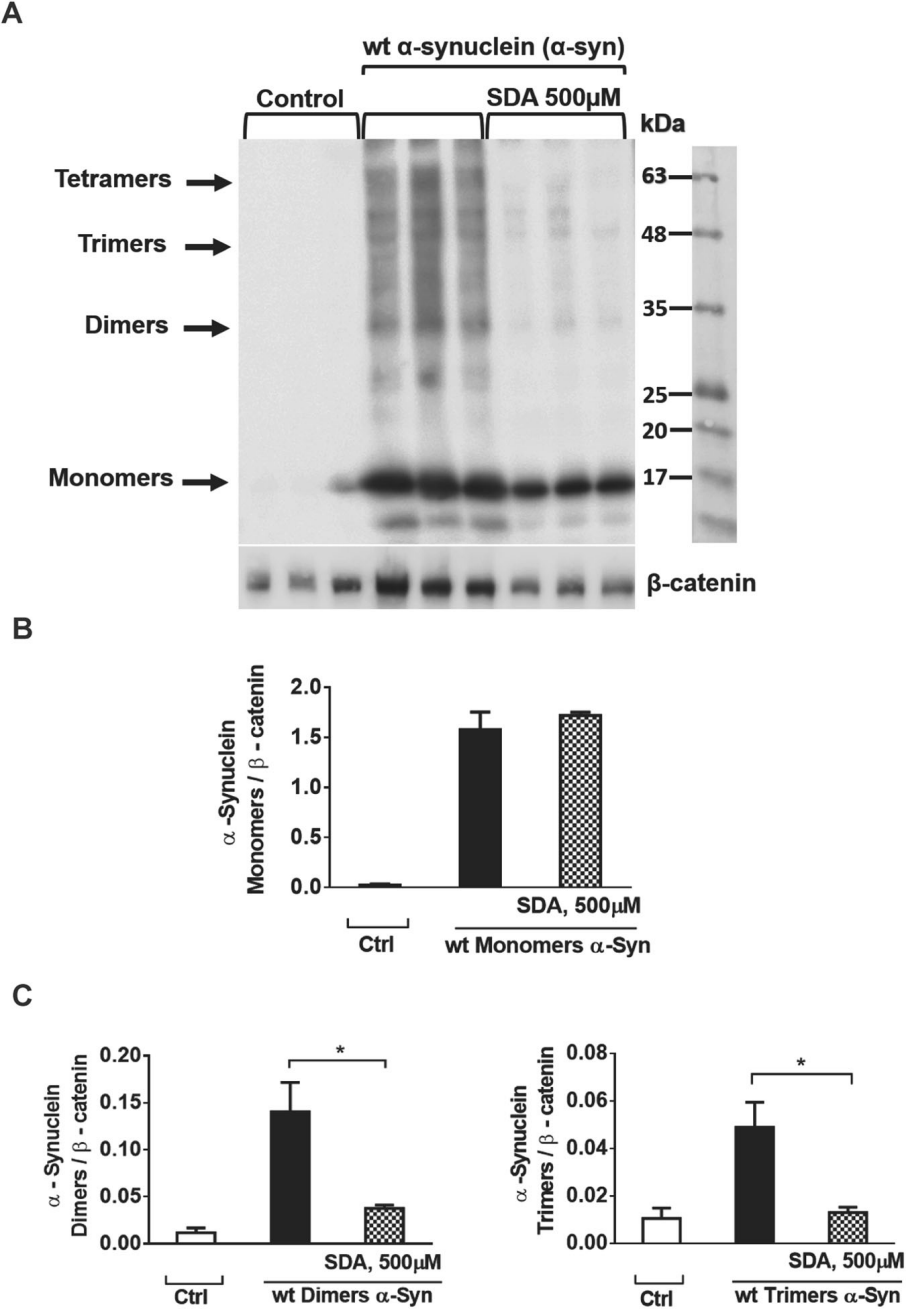
SDA lower accumulation of dimer and trimer forms of α-synuclein in HEK293 overexpressing α-syn. HEK293 cells were transfected with wt α-syn, and 24 h later washed, and treated with or without 500 μM SDA and incubated for additional 24 h. The cells were lysed 48 h after transfection and heated to 65 °C overnight. **A** Equal amounts of protein were loaded and separated on 12% SDS-PAGE. α-syn was determined by immunoblotting using the corresponding α-syn antibody. Control represents non-transfected cells. **B** Quantification of α-syn monomers normalized to β-catenin. **C** Quantification of α-syn dimers and trimers normalized to β-catenin. The amount of each band was quantitated by densitometry and plotted with a linear regression program. The values shown are average (±SEM) of an experiment consisting of triplicates normalized to β-catenin.

The efficacy of SDA in preventing α-syn aggregation was tested also in HEK293 overexpressing α-syn. HEK293 cells were transfected with cDNA encoding wt α-syn. Forty-eight hours after transfection, the cells exhibited high levels of monomers, and aggregated forms of α-syn, monitored by the corresponding α-syn antibodies (Fig. 8A) (see Fig. S13). Preincubation of α-syn transfected HEK293 cells with 500 μM SDA showed a significant decrease in dimers and trimers of α-syn, with no apparent change in the expression of the monomeric forms of α-syn (Fig. 8A–C). The mechanism of lowering levels of aggregated forms of α-syn by SDA remains to be understood.

## Discussion

Slowing the progression of PD remains a critical need in the therapy of the disease. Levodopa, the presently used treatment like dopamine itself, is known to form toxic semiquinones by auto‐oxidation. This in turn leads to a decrease in GSH level and cell death (review [37]).

In the present study our goal is to characterize two novel levodopa precursors comprising of anti-inflammatory/anti-apoptotic activities, which may well protect DA neurons from neuroinflammation and premature cell death. Small molecular weight levodopa analogs were prepared, in which the antioxidant NAC is linked via a peptide bond to either the N-terminal of L-DOPA-Cys-amide, forming SD or to the N-terminal of L-DOPA amide, forming SDA. Both SD and SDA were designed to provide NAC and Cys into nigral DA cells concomitantly with L-DOPA delivery, aimed at replenishing the missing dopamine simultaneously with reversing oxidative stress/inflammatory damages. Treatment with SD and SDA having multifaceted activities combined into a single molecule have the potential to delay the onset of both motor fluctuations and dyskinesias and slow premature loss of nigrostriatal denervation of the dorsal putamen and nigral cells.

### Rotenone rat model of Parkinson’s disease (PD); In vivo studies

To assess the neuroprotective properties of SD and SDA in vivo, we used the systemic acute rotenone rat model that reproducibly mimics many aspects of the pathology of human PD [32]. In this model, the i.p injection of 3.0 mg/kg/day rotenone produces dopamine terminal loss in the dorsolateral striatum, a selective nigrostriatal degeneration, loss of tyrosine hydroxylase positive neurons without nonspecific lesions, as well as induction of α-syn and poly-ubiquitin aggregation [32, 38].

Since the rotenone-treated animals develop bradykinesia, postural instability, and/or rigidity [32], this model serves as an excellent tool to test novel neuroprotective reagents that could provide insights into the pathogenesis of PD.

Motor coordination and balance were monitored in the rotenone/only, rotenone/SD and rotenone/SDA treated rats, using rotarod, beam walking, and rearing activity. Postural instability, and/or rigidity were developed by all rotenone/only-treated animals, and reversed in the rotenone/SD- or rotenone/SDA-treated rats. The significant rescue of impaired motor activities subsequent to intraperitoneal injection strongly suggests that both SD and SDA may well cross the BBB, reversing the rotenone-triggered toxicity of the DA neurons. The intraperitoneal injection of the TXM-peptide, TXM-CB3, was previously shown to reverse inflammatory consequences in the brain of diabetes Zucker rats [24]. These results support a potential anti-inflammatory activity of SD, the newly designed member of the TXM-peptide family, and its ability to cross the BBB which might help managing PD motor impairment.

### Anti-inflammatory and anti-apoptotic activity of SD and SDA; In vitro studies

It is well established that the inflammatory/apoptotic pathway is activated by the apoptosis signal-regulating kinase1 (ASK1) [39]. ASK1 forms a stable complex with reduced thioredoxin 1 (Trx1_red_). This ASK1/Trx1_red_ complex is unable to activate the MAPK-apoptotic pathway. Upon oxidation of Trx1_red_ to Trx1_ox_, the complex dissociates enabling free ASK1 to activate the MAPK-inflammatory/apoptotic pathway.

The anti-inflammatory/antioxidant properties of SD and SDA have been demonstrated in vitro, monitoring the activation of MAPK pathway in the neuronal human neuroblastoma SH-SY5Y cells. Oxidative stress was induced in the cells by Auf, an organogold selective inhibitor of thioredoxin reductase, known to activate ASK1 by maintaining Trx1 in the oxidized form. Through the activation of the MAPK-inflammatory/apoptotic pathway, Auf also inhibits the mitochondrial Trx2 causing the failure of mitochondrial function in the brain [40]. Furthermore, Auf inhibits mitophagic flux, lowering MMP and ATP levels [35].

The ability of SD and SDA to reverse the Auf-oxidizing effects implies a dual mechanism of action. SD and SDA exert an indirect antioxidant effect by reducing Trx1_ox_ to Trx1_red_ with resultant activation of ASK1, and/or a direct effect of ROS scavenging and elevation of GSH levels. These effects establish the anti-apoptotic/anti-inflammatory activities of SD and SDA in neuronal cells, promoting potential clinical relevance in protecting nigrostriatal denervation of the dorsal putamen and slowing nigral cell loss.

### Combined therapies in a single molecule

The in vivo studies suggest that SD or SDA, which have both NAC and levodopa moieties within them, appear to cross the BBB, thereby facilitating delivery of both components to the targeted cells. This is in contrast to NAC which is BBB-impermeable. Acting as a vehicle to bring NAC into the brain, SD and SDA might provide antioxidant protection to DA neurons, simultaneously with replenishing the missing dopamine in the nigral cells. The data suggest that the combined activities of SD and SDA into a single molecule, might have a significant advantage over NAC and levodopa administrated separately. In the absence of a clinically efficient neuroprotective treatment to slow PD progression, SD and SDA might have a putative disease-modifying effects by conferring antioxidant/anti-apoptotic activity within the DA neurons.

### Preventing α-syn aggregation

Alpha-synuclein (α-syn) is abundantly expressed in neurons and is the major constituent of Lewy bodies, which are the hallmarks of neurodegenerative diseases called synucleinopathies, which like PD, results in multiple system atrophy, Lewy body dementia, Alzheimer’s disease, and frontotemporal dementia.

The prevalence of misfolded fibrillar aggregates of α-syn associated with Lewy bodies is consistent with the role of copper ions and oxidative stress, forming highly toxic α-syn oligomers and α-syn aggregation [41]. Copper ions are elevated in the cerebrospinal fluid of PD patients and linked to α-syn oligomerization [18, 42–44].

Since sulfhydryl groups are known to chelate copper ions, SD and SDA were examine for the effects on α-syn expression and aggregation.

Both SD and SDA showed a reduction in α-syn expression in SH-SY5Y cells exposed to a high dose of rotenone. Also, production of aggregated forms of α-syn was significantly lower in HEK293 cells overexpressing α-syn in the presence of SDA. The precise mechanism by which SD or SDA prevent stress-induced α-syn expression in neuronal cells is not yet clear and requires further study.

The second experiment investigated a direct effect of SDA on α-syn oligomerization, using α-syn transiently transfected HEK293 cells, which express high levels of α-syn monomers and oligomers [45]. Preincubation of the cells with SDA, resulted in a significantly lower level of α-syn dimers and trimers, but no change in the level of α-syn monomers. This experiment implies SDA interference in α-syn oligomerization, possibly by copper ions-chelating activity. Because α-syn transfected HEK293 cells do not represent the complexity and morphological features of α-syn inclusions or Lewy bodies in neuronal cells, further studies are required to establish the relevance lowering α-syn oligomerization to DA neurons.

## Conclusions

The newly designed levodopa precursors, SD and SDA appear to relieve motor impairment in the rotenone rat model and inhibit oxidative stress-induced activation of the inflammatory MAPK-apoptotic pathway in neuronal cells. Initial data also suggest that SDA reverses α-syn oligomerization.

To date, there is no clinically disease-modifying treatment that can halt PD progression. The failure to develop neuroprotective or disease-modifying strategies results mainly because treatment starts with the appearance of motor symptoms, long after a significant nigral cell loss. Nevertheless, the standard treatment of levodopa, which is almost entirely centered on dopamine replenishment performs rather efficiently during the initial 4–5 years of treatment, implying viability of the remaining DA neurons. Therefore replacing levodopa treatment with SD or SDA may possibly attenuate the loss of the remaining nigral cells and slow PD progression. Future toxicology studies and subsequent clinical testing of SD or SDA alongside levodopa could establish their potential clinical benefits and advantage in halting disease progression.

The combined redox activity and levodopa release with lowering α-syn aggregation might potentially slow-down nigral cell loss, protect nigrostriatal denervation of the dorsal putamen, and attenuate Lewy pathology. Hence, SD and SDA have the potential to become a disease-modifying treatment of PD or other LB-like pathologies.

## Methods

### Materials

Auranofin (Enzo Life Sciences, Shoham, Israel), triethylphosphine (2,3,4,6-tetra-O-acetyl-β-1-d-thiopyranosato-S) gold(I); LDEE from TEVA Ltd Israel. SD, SDA, and TXM-peptide TXM-CB3, and AD4 (NAC-amide), were custom synthesized by Novetide, Ltd, Haifa, Israel. All other materials were purchased from Sigma, Jerusalem. α-syn plasmid was a kind gift of Dr. R. Sharon (Hadassah Ein Kerem, Jerusalem).

### Cell culture and treatment

Human neuroblastoma SH-SY5Y cells were cultured in DMEM/F12 HAM 1:1 medium supplemented with 10% fetal bovine serum (FBS) and penicillin–streptomycin, incubated at 37 °C with 5% CO_2_, as previously reported [24]. Human embryonic kidney HEK293 cells were cultured at 37 °C, 5% CO_2_ in RPMI 1640 supplemented with: l-alanyl-l-glutamine (4.4 mM); 10% fetal bovine serum; penicillin (100 U/ml) and streptomycin (100 μg/ml); HEPES pH 7.3 (10 mM). Cells were plated at a density of 6.25 × 10^4^/cm^2^ and incubated for 24 h, after which they were exposed to different treatments. Tissue culture serum and medium were from Biological Industries (Kibbutz Beit-Haemek, Israel).

### In vitro studies using SH-SY5Y cells

The anti-inflammatory activity of SD and SDA was tested essentially as previously reported for TXM-CB3 [24]. SH-SY5Y cells were incubated for 30 min with a 3 μM auranofin (AuF), washed and incubated for 3.5 h with or without SD, SDA, TXM-CB3, at the indicated concentrations. After washing with one ml of PBS the cells were lysed in 0.12 ml lysis buffer (150 mM Tris, pH 6.8, 10% Glycerol, 0.6% SDS, Bromophenol Blue, supplemented with 7 μl β-Mercaptoethanol/ml). Protein concentration of the lysates was determined by using Coomassie Brilliant-Blue staining. Cell lysates were heated to 100 °C for 10 min prior to electrophoresis separation on SDS-PAGE gels.

### Western blot analysis and antibodies

Western blot analysis were performed essentially as previously published [23]. Twenty to thirty micrograms of protein samples were loaded on 10 or 12% SDS-PAGE gels. The proteins were then transferred electrophoretically to nitrocellulose (Whatman, Germany). The blots were cut prior to hybridization with antibodies, and blocked by incubation for 1 h at RT in TBS-T (25 mM Tris–HCl pH 7.4, 0.9% NaCl, and 0.02% Tween-20) with 4% Difco skim milk (BD, USA), and incubated overnight at 4 °C with the primary antibody: pERK1/2 (Thr 202/Tyr204), mouse mAb; ERK2 (Santa Cruz, USA) rabbit Ab; p-SAPK/JNK (Thr183/Tyr185), rabbit mAb; SAPK/JNK, mouse mAb; p-p38^MAPK^ (Thr180/Tyr182), rabbit mAb; p38, rabbit Ab; GAPDH (Glyceraldehyde 3-phosphate dehydrogenase); α-syn, Abcam, Cambridge, UK EPR20535; β Catenin, mouse mAb (1:10,000; BD Transduction Laboratories, USA) diluted in 5% BSA, 0.04% Azide in TBS-T. Proteins were detected with Anti-Mouse or Anti-Rabbit IgG-HRP linked antibody (1:10,000; Cell Signaling Tech., USA).

### Alpha-synuclein (α-syn) aggregation in transfected HEK293 cells

HEK293 cells were plated on collagen (rat tail) (Roche Diagnostics, Mannheim, Germany) and incubated for 16 h in 24-well plates and allowed to reach 70–80% confluency in 1 ml of culture medium. The next day, the cells were washed twice with regular DMEM, incubated at 37 °C, for 5 h in Opti MEM (Thermo Fisher Scientific, Waltham, MA) containing the transfection mixtures of 0.75 μg plasmid cDNA encoding wt α-syn and polyethyleneimine (PEI) at a ratio of 1:3 (DNA to PEI) in 400 µl Opti MEM after vortex for 10 s, and incubated at RT for 15 min. After 5 h the medium was replaced by regular DMEM containing 10% FBS solution. Cell media was replaced after overnight incubation, with DMEM containing 10% FBS solution with or w/o 500 µM of SDA and incubated for 48 h. Then the cells were lysed with 0.12 ml ice-cold lysis buffer (150 mM Tris–HCl, pH 6.8), 10% glycerol, 0.6% SDS, bromophenol blue, supplemented with 7 μl β-mercaptoethanol, and heated overnight at 65 °C. Proteins were separated on SDS-PAGE α-syn monomers, dimers, and trimers were detected after blotting to nitrocellulose with the corresponding α-syn antibodies.

### Induction of α-synuclein by rotenone in SH-SY5Y cells

SH-SY5Y cells were treated with increasing concentration of SD or SDA for 60 min. Then the cells were washed and incubated with 5 µM rotenone for 16 h. The level of α-synuclein in cell lysates was determined after protein separation on 12% SDS-PAGE using α-syn antibodies. The values calculated by densitometry shown as averages (±SEM) of two independent experiments normalized with housekeeping level of β-catenin; Student’s t-test (two populations) was performed for rotenone-treated cells. *P value < 0.05; **P value < 0.01.

### Animals

#### Ethics

A commercial company Science in Action, Nes-Ziona, Israel, uses its ethical permission to do animal studies as an outsourcing service (#C148210).

All animals were treated according to the National Institute of Health (NIH) guidelines for the care and use of laboratory animals. Animal ethics committee accredits the company, and licensed veterinarians conducted the experiments. Rat were sacrificed by an overdose of CO_2_ and decapitated.

### Rotenone treatment

The experiments were performed by “Science in Action” Rehovot, according to Cannon et al. [32]. Rotenone (Sigma, St Lewis, MO, USA) was dissolved (3 mg/ml) in DMSO (10%w/in sunflower oil pH 7.4). During the treatment period (10 days) rotenone was stored at 6 ^o^C. Twenty male Sprague Dawley rats 7-8 weeks (315 g; Envigo, Rehovot) were divided into 4 experimental groups. The four groups comprised of rotenone only (*n* = 6), rotenone with SD (33 mg/kg) (*n* = 6), rotenone with SDA (33 mg/kg) (*n* = 6), and naive rats (*n* = 2) that were not treated and kept under the same conditions throughout the experiment. SD and SDA were administered intraperitoneally (i.p.) once a day in the afternoon (days 1–9).

### Animal behavior

Animals body weight was determined before initiation of treatment (day 0), on days 4, 7, 10 during the experiment and on termination day 11. On day 11 the animals were sacrificed.

Rearing behavior, rotarod, and beam walk tests were performed before initiation of treatment (day 0) and on days 4, 7, and 10 of the experiment.

### Rotarod behavior test

Motor coordination and balance was evaluated on the rotarod, which was set to accelerate from 4 to 40 rpm in 300 sec. Animals were placed at separate lanes on the rotarod with initial rotation set on 4 rpm.

### Rat-rearing behavior test

Animals were placed in a clear glass cylinder (40 cm high × 20 cm diameter) and number of rears in 2 min was monitored. Rear was considered when animals raised their front legs above the shoulder and made a contact with the wall of the cylinder with their forelimb.

### Rat beam walk

Animals were trained for 2 days to traverse the length of the beam. On the day of the test animals were gently placed on the 1 m long narrow aluminum beam facing one of the ends and allowed to walk along the beam, monitoring the time taken to reach the end of the beam.

### Statistical analysis

All values were presented as mean ± standard deviation (SD) or standard error of mean (SEM), and differences were considered to be statistically significant at the *P < 0.05 level [31]. Statistical analysis was performed using StatsDirect statistical software (Cheshire, UK). Differences among means were analyzed using one‐way ANOVA followed by comparison to the control rotenone-only‐treated rats group (Dunnet’s test). Within groups, comparison to the baseline was performed by two‐way ANOVA, and nonparametric data were analyzed with Kruskal–Wallis ANOVA or Friedman ANOVA, respectively.

## Supplementary information

Marked-up manuscript

Supplemental Material

## Data Availability

All data generated or analyzed during this study are included in this published article.
