# Supplementary material for: Novel anti-apoptotic L-DOPA precursors SuperDopa and SuperDopamide as potential neuroprotective agents for halting/delaying progression of Parkinson’s disease
Source: Cell Death Dis. 2022 Mar 11;13(3):227. doi: 10.1038/s41419-022-04667-2 (PMC8917195; doi:10.1038/s41419-022-04667-2)
Supplement: Supplementary file 1 — Marked-up manuscript [file 41419_2022_4667_MOESM1_ESM.docx]

**Novel** **Anti-apoptotic L-DOPA Precursors SuperDopa and SuperDopamide as Potential Neuroprotective Agents for Halting/Delaying** **Progression of Parkinson’s Disease**

**Tom Wiesen, and Daphne Atlas*** https://orcid.org/ **0000-0002-5160-722X**

daphne.atlas@mail.huji.ac.il

Dept. of Biological Chemistry

Institute of Life Sciences

The Hebrew University of Jerusalem

Jerusalem, 91904 Israel

**Key words:** Neurodegeneration; Inflammation; α-synuclein; Oxidative stress; L-dopa precursors; Apoptosis; MAPK; Thioredoxin-mimetic-peptides

**Running title:** Anti-apoptotic/anti-inflammatory L-DOPA Precursors

**Work count**: 3677

**References count**: 45

***Correspondence**

Daphne Atlas, MSc, Ph.D.

Professor of Neurochemistry

Dept. of Biological Chemistry Institute of Life Sciences

The Hebrew University of Jerusalem

Jerusalem, 91904 Israel

Phone 972-2-658-5406 Fax 972-2-651-2958

daphne.atlas@mail.huji.ac.il

**Abstract**

Parkinson’s disease (PD) is characterized by a gradual degeneration of the dopaminergic neurons in the substantia nigra pars-compacta (SNpC). Levodopa, the standard PD treatment, provides the missing dopamine in SNpC, but ultimately after a honeymoon with levodopa treatment the neurodegenerative process and the progression of the disease continue.

Aimed at prolonging the life of dopaminergic cells, we prepared the levodopa precursors SuperDopa (SD) and SueprDopamide (SDA), in which levodopa is merged with the antioxidant N-acetylcysteine (NAC) into a single molecule.

Rotenone is a mitochondrial complex inhibitor is often used as experimental model of PD. *In vivo,* SD and SDA treatment show a significant relief of motor disabilities in rotenone-injected rats. SD and SDA also lower rotenone-induced-α-synuclein (α-syn) expression in human SH-SY5Y cells, and α-syn oligomerization in α-syn-overexpressing-HEK293 cells. In the neuronal SH-SY5Y cells, SD and SDA reverse oxidative stress-induced phosphorylation of cJun-N-terminal kinase (JNK) and p38-mitogen-activated kinase (p38^MAPK^).

Attenuation of the MAPK-inflammatory/apoptotic pathway in SH-SY5Y cells concurrent with protection of rotenone-triggered motor impairment in rats, is a manifestation of the combined antioxidant/anti-inflammatory activity of SD and SDA together with levodopa release.

The concept of joined therapies into a single molecule, where levodopa precursors confer antioxidant activity by enabling NAC delivery across the BBB, provides a potential disease-modifying treatment for slowing PD progression.

**Background**

Neurodegenerative diseases share neuroinflammation as a common mechanism resulting from activated microglia that release pro-inflammatory cytokines. The loss of 9-type dopaminergic (DA) neurons in the substantia-nigra pars compacta (SNpc) has been shown to be responsible for the first motor symptoms of Parkinson’s disease (PD).

Virtually all PD patients will require levodopa therapy. However, PD progresses with time, and patients experience diminished duration of benefit from each dose. The diminished effectiveness over time is also accompanied by the development of medication-related complications such as motor fluctuations, and levodopa-induced dyskinesia (LID) (1). The decrease in efficacy of levodopa is attributed mainly to the continuous loss of DA cells, presenting an unmet medical need with no approved drug therapy. It requires a disease-modifying approaches that would delay or slow the clinical progression of the disease (reviews (1), (2), (3)).

Although the cause of neurodegeneration processes in sporadic PD is not fully understood, constitutive production of reactive oxygen species (ROS) during the oxidative metabolism of dopamine within the DA cells, and impaired bioenergetics in the mitochondria contribute to premature cell death. Oxidative stress also triggers the mitogen-activated protein kinases (MAPKs) inflammatory/apoptotic pathways through the phosphorylation of c-Jun N-terminal kinase (JNK) and p38 mitogen protein kinase (p38^MAPK^) (review (4)), (5), (6, 7).

Mitochondrial toxins including herbicide paraquat, pesticide rotenone, or the funcocide MB-manganese, target a variety of redox sensitive proteins in the brain contributing to sporadic PD (8). These toxins induce mitochondrial electron-transport chain dysfunction, mainly through complex1 inhibition, and are often used as disease models for PD. Similar to these environmental mitochondrial toxins, PD-linked gene mutations have been shown to be associated with mitochondrial damage combined with oxidative/nitrosative stress (9). An increase in the basal and mitochondrial toxin-induced nitrosative stress resulted in inhibition of transcriptional activity of a critical cysteine residue in myocyte-specific enhancer factor 2C (MEF2C), which is a redox-mediated protein that enhances DA neurons in alpha-synuclein mutant (α-syn^A53T^) A9 DA neurons (10).

Oxidative/nitrosative stress induced by paraquat has been shown to modify α-syn by nitration of a tyrosine residue and oxidation of a methionine residue, thereby contributing to its aggregation (11) (12), (13). These results are consistent with the identification of nitrosative/oxidative stress as one of the players in α-syn aggregation, and provide a molecular link to the cascade of events leading to the selective death of SNpc DA neurons in PD. The accumulation of α-syn may also impair mitochondrial homeostasis by decreasing the activity of mitochondrial complex1 (14). The wt α-syn and the mutated α-syn^A53T^ are linked to genetic mutations that generate intracellular inclusions called Lewy body that contribute to the neuronal dysfunction and pathology of DA cells (15), (16), (17). These aberrant protein aggregations are one of PD hallmark and are associated with death of dopamine-producing cells (18), (19), (20).

To develop a disease modifying strategy for PD, we designed two dopamine precursors, AcCys-L-Dopa-Cys amide, called SuperDopa (SD) and AcCys-L-Dopa amide, called SuperDopAmide (SDA).

SD is a family member of the thioredoxin mimetic peptides (TXM-peptides). TXM- peptides protect neuronal and non-neuronal cells from apoptosis *in vitro* and *in vivo,* by inhibiting the oxidative stress-induced MAPK inflammatory/apoptotic pathway and catalyzing S-denitrosylation (7), (21), (22), (23), (24), (25), (26), (27), (28), (29), (30).

SDA is a dipeptide comprising of N-acetylcysteine (NAC) and DopAmide. DopAmide itself is a levodopa precursor that confers a sustained release of dopamine in 6-OH-dopamine-lesioned rats (31).

To recapitulate *in vivo* features of SD and SDA we used the highly reproducible rotenone-rat-model, in which rotenone-treated rats develop PD features like bradykinesia, postural instability and/or rigidity, thus providing an excellent tool to test potential PD neuroprotective reagents (32). *In vitro,* SD and SDA effects on the antiapoptotic/anti-inflammatory activity and on α-syn aggregation were investigated in human neuroblastoma SH-SY5Y cells and in HEK293 overexpressing α-syn.

As demonstrated, both SD and SDA appeared to combine anti-inflammatory/antioxidant activities with the ability to replenish the cells with levodopa. The impact of joined activities into a single molecule is often greater than administrating each molecule separately.

The concept of combined therapies of levodopa precursor with antioxidant activity into a single molecule that enables NAC delivery across the BBB and inhibition of the neurodegenerative process, could become a disease-modifying treatment with an outlook for slowing PD progression.

**Results**

**Design and synthesis of SuperDopa (SD)**

SuperDopa (AcCys-L-DOPA-Cys-amide; SD), is N-acetylated tri-peptide comprising of two cysteine (Cys) residues that flank levodopa (**Fig. 1; *upper***). SD was synthesized (Novetide, Ltd) and its purity (98%) and molecular weight were determined by HPLC (**Fig. S1)**, and mass spectra (**Fig. S2)**. The acetylation at the amino-terminal and the α-C-amidation of SD, neutralize the positive and negative charges of the peptide. These modifications increase lipophilicity and lead to enhanced membrane targeting and cell membrane permeation. SD is structurally analogous to the thioredoxin mimetic (TXM) peptide, TXM-CB3 (Ac-Cys-Pro-Cys-amide) that mimics thioredoxin activity (21), (23), (22), (26), (27), (28). As a peptide, SD is susceptible to cleavage by endo-and amino-peptidases (**Fig 1; *upper; red arrows***). The intracellular proteolysis of SD generates levodopa, NAC, and Cys-amide. NAC and Cys-amide are hydrolyzed further, to yield two Cys residues. Both NAC and Cys are reducing regents known as glutathione (GSH) precursors. In addition, cleavage of SD releases levodopa. The non-charged SD, similar to TXM-CB3, is predicted to cross the BBB (24), enabling transport of levodopa, NAC, and Cys across the BBB onto dopaminergic neurons.

**Design and synthesis of SuperDopAmide (SDA)**

SuperDopAmide (N-Acetylcysteine-L-DOPA-amide; SDA), is an N-acetyl blocked dipeptide comprising of a single Cys and a single residue of levodopa modified at the carboxy-terminal to an amide. SDA was synthesized (Novetide, Ltd), and its purity (98%) and molecular weight were determined by HPLC (**Fig. S3)**, and mass spectra (**Fig. S4)**. SDA proteolysis (**Fig. 1; *lower; red arrow***) generates NAC, and levodopa-amide (DopAmide) (**Fig. 1; *lower, red arrow***). DopAmide itself is a levodopa precursor, which is hydrolyzed to levodopa and replenishes cellular dopamine (31). The multiple activities of SDA, NAC and elevating levodopa, are predicted to provide neuroprotection simultaneously with increasing dopamine levels.

***In vivo* studies**

Here, SD and SD*,* were tested *in vivo* using the rotenone rat-model, which mimics PD motor dysregulations and is one of the most reliable animal models of PD. Three groups of rats were injected intraperitoneally (i.p) in the morning with rotenone (3.0 mg/kg/day) in a specialized vehicle, for 9 days. In the afternoon, one group was i.p injected with SDA (33 mg/kg/day) and another group, with SD (33 mg/kg/day), for 9 days (**Materials and Methods**).

Body weight was monitored on days 4, 7, and 10 (**Fig. 2A**). A slight reduction in body weight in rotenone/only injected rats was observed, compared to naïve rats, and no significant decrease was observed in rats treated with rotenone/SD or rotenone/SDA, as quantified on day 10 (**Fig. 2B**). The rotenone/only-treated rats developed bradykinesia, postural instability, and/or rigidity, which were not observed in the rotenone/SD and rotenone/SDA treated-rats.

***SD and SDA prevent motor impairment in the rotenone rat model***

***Rotarod behavior test*** Motor coordination and balance were evaluated using the rotarod behavior test at days 4, 7, and 10 (**Fig. 3A** *left*). The behavioral performance of treated rats compared to naïve rats was quantified on day 10 (**Fig. 3A**, *right*). A statistically significant difference in performance was observed between rotenone-treated rats and naïve rats. In sharp contrast, rats treated with rotenone together with SDA or SD, showed longer timing on the rotarod, indicating a significant protection of motor activity, compared to rotenone only/treated rats. Performance on rotarod of rotenone/SD or rotenone/SDA treated rats was similar to naïve rats.

***Rat-rearing behavior test*** (*Cylinder assay*) The rearing cylinder test was used to evaluate locomotor activity in rodent models of CNS disorders. A gradual decrease in the ability of the rotenone-treated rats to place the palm on the wall of the cylinder was monitored at days 4, 7, and 10, indicating impaired body support (**Fig. 3B** *left*). Rats treated with rotenone/SD or rotenone/SD displayed rearing activity similar to naïve rats, showing a significant improvement of rearing activity. The performance of rearing activity was quantified on day 10 (**Fig. 3B** *right*).

**Rat beam walk test** was applied for examining SDA and SD effects on motor coordination and balance in the rotenone rat model. The goal of this assay was to test the rat ability to stay upright and walk across an elevated narrow beam to a safe platform. After two days training, the assay examined the time it takes for the rat to traverse the beam and the number of paw slips that occur in the process. This test is complementary to the rotarod, and can detect subtle deficits in motor skills and balance.

A significant improvement in the time required for terminating the walk was observed in the rotenone/SD or rotenone/SDA treated rats, compared to rotenone/only, at days 4, 7, and 10 (**Fig. 3C** *left*). Quantified at day10, the rotenone/only treated rats displayed a significant longer time to complete the beam walk compared to rotenone/SDA or rotenone/SD treated rats, (**Fig. 3C,** *right*)*.* The central nervous system mediated locomotor activity, which was protected subsequent to i.p injection of SD and SDA, indicates BBB permeation of both compounds.

***In vitro* studies**

To assess a putative neuroprotective mechanism, and cell permeation we examined the antioxidant/anti-inflammatory activities of SD and SDA, by monitoring the oxidative stress-induced MAPK-apoptotic/inflammatory pathway in human neuroblastoma SH-SY5Y cells. Oxidative stress was induced by auranofin (Auf), an inhibitor of thioredoxin reductase. SH-SY5Y cells are often used as a cellular model of various neurological oxidative stress-related disorders such as PD and Alzheimer’s disease (33), (34).

**Mitochondrial membrane potential**

Initially, we explored the effects of Auf on the mitochondria, recording mitochondrial membrane potential. Similar to rotenone and paraquat, we found that Auf lowered the mitochondrial membrane potential (MMP; **Fig. S5**). These results also confirm previous studies showing mitochondrial dysfunction by Auf (35).

**SD, SDA, and TXM-CB3 reverse the Auf-induced morphological changes in SH-SY5Y cells**

Next, we wanted to assess the effects of SD and SDA on Auf- or rotenone-induced cell morphology. SH-SY5Y were incubated with 3 µM Auf for 30 min, washed, and incubated for 3.5 h at 37^o^C with or without SD or SDA. As shown in **Fig. S6**, morphological changes, appeared 4 hr after Auf-treatment, and were accompanied with a reduction in cell number, and a loss of cell-to-cell contact. These changes were reversed in cells incubated with either SD, SDA, or TXM-CB3 (**Fig. S6**). No change however, was observed in cell morphology in cells incubated with 5µM rotenone for 3.5 h at 37^o^C (**Fig. S7**). Previous studies quantified the Auf effects on cell viability and early and late apoptosis (23).

**SD and SDA reverse Auf-induced JNK phosphorylation in SH-SY5Y cells**

Next, to investigate the possible anti-apoptotic/anti-inflammatory activity of SD and SDA we explored Auf-induced JNK phosphorylation in SH-SY5Y cells. The cells were treated with 3μM Auf for 30 min, washed, and then incubated for 3.5 h at 37^o^C with or without SD (**Fig. 4A**) or SDA (**Fig. 4B**) (see **Fig. S8**), at the indicated concentrations. JNK1/2 phosphorylation was monitored by western blot analysis using the anti-phospho JNK1 antibodies, and normalized to total JNK1 or β-catenin, with the corresponding anti-JNK1 or anti-β-catenin antibodies. The reduction in Auf-induced JNK phosphorylation mediated by SD or SDA was concentration-dependent with apparent dissociation constant of Ki=50.2 ± 1.1 µM, and Ki=14.2± 1.4 µM, respectively. Hence, by inhibiting JNK1 phosphorylation, SD and SDA inhibit the MAPK-inflammatory/apoptotic pathway.

**SD and SDA reverse Auf-induced p38^MAPK^ phosphorylation**

Next, the ability of SD and SDA to reverse Auf-induced p38^MAPK^ phosphorylation was examined in SH-SY5Y cells. The cells were treated for 30 min with 3μM Auf, washed, and incubated for 3.5 h at 37^o^C, with or without SD (**Fig. 5A**) or SDA (**Fig. 5B**) (see **Fig. S9**), at the indicated concentrations. Phosphorylation of p38^MAPK^ was monitored by western blot analysis using anti-phospho-p38^MAPK^ antibodies and normalized to β-catenin using anti β-catenin antibodies. The reduction in Auf-induced p38^MAPK^ phosphorylation mediated by SD and SDA was concentration-dependent, displaying an apparent dissociation constant Ki =17.9 ± 1.2 µM, and Ki =11.2 ± 1.4 µM respectively.

Hence, SD and SDA appear to reverse Auf-induced apoptosis through the ASK1-MEK/JNK or ASK1-MEK/p38^MAPK^ pathways. Similar anti-apoptotic activity of a variety of other TXM-peptides was previously reported (23), (24), (21).

**The effect of SD on Auf-induced ERK1/2 phosphorylation**

As opposed to JNK and p38^MAPK^, SD showed no significant decrease in Auf-triggered phosphorylation of the Extracellular signal Regulated Kinases (ERK1/2) (**Fig. S10**). This result is consistent with a marginal reversal of the Auf-induced ERK1/2 phosphorylation, previously reported for the thioredoxin mimetic peptide TXM-CB3 (23), (24).

**Levodopa ethyl ester (LDEE) does not reverse oxidative stress induced activation of JNK or p38 apoptotic pathway**

The antioxidant activity of SD and SDA was compared to another levodopa precursor, levodopa ethyl ester (LDEE) (36), testing the reversal of Auf-induced JNK, and p38^MAPK^ phosphorylation in SH-SY5Y cells. The cells were treated for 30 min with 3μM Auf, washed, and then incubated for 3.5 h at 37^o^C, with or without increasing concentrations of LDEE (**Fig. S11**). As opposed to SD and SDA, LDEE did not reverse the Auf-induced phosphorylation of JNK or p38^MAPK^ at concentrations up to 200µM (**Fig.** **S11)**.

The effects of the three precursors SD, SDA, and LDEE, on Auf-induced JNK and p38^MAPK^ phosphorylation are compared (**Fig. 6)**. SD and SDA but not LDEE, exhibited a concentration dependent decrease in Auf-induced phosphorylation of JNK and p38^MAPK^ (**Fig. 6A,B**). These findings show the antioxidant activity of SD and SDA, which could be attributed to the thiol group of the Cys residue, most likely through preventing the dissociation of Trx/ASK1 (23).

**The effects of SD and SDA on rotenone-induced expression of α-synuclein (α-syn)**

Copper ions play a role in synucleopaties, while the Cys-sulfhydryl group are known as copper chelators. These activities led us to investigate the effect of SD or SDA on rotenone-induced expression of α-syn in SH-SY5Y cells. Cells treated for 60 min with SDA (**Fig. 7A**) or SD (**Fig. 7B**) at the indicated concentrations, were washed, and then incubated with 5µM rotenone for 24 h. The expression of α-syn was determined by western blot analysis, using α-syn antibodies normalized to β-catenin. Incubation of the cells with rotenone triggered α-syn expression, was significantly higher than in untreated cells, and markedly suppressed in 10µM of SD or 10µM SDA treated-cells (see **Fig. S12)**

**SDA lowers α-synuclein (α-syn) aggregation**

The efficacy of SDA in preventing α-syn aggregation was tested also in HEK293 overexpressing α-syn. HEK293 cells were transfected with cDNA encoding wt α-syn. Forty eight hr after transfection, the cells exhibited high levels of monomers, and aggregated forms of α-syn, monitored by the corresponding α-syn antibodies (**Fig. 8A)** (see **Fig. S13)**. Preincubation of α-syn transfected-HEK293 cells with 500μM SDA showed a significant decrease in dimers and trimers of α-syn, with no apparent change in the expression of the monomeric forms of α-syn (**Fig. 8A,B,C**). The mechanism of lowering levels of aggregated forms of α-syn by SDA remains to be understood.

**Discussion**

Slowing the progression of PD remains a critical need in the therapy of the disease. Levodopa, the presently used treatment like dopamine itself, is known to form toxic semiquinones by auto‐oxidation. This in turn leads to a decrease in GSH level and cell death )Review (37)).

In the present study our goal is to characterize two novel levodopa precursors comprising of anti-inflammatory/antiapoptotic activities, which may well protect dopaminergic neurons from neuroinflammation and premature cell death. Small molecular weight levodopa analogues were prepared, in which the antioxidant NAC is linked via a peptide bond to either L-DOPA-Cys-amide, forming SD or to L-DOPA amide, forming SDA. Both SD and SDA were designed to provide NAC and Cys into nigral dopaminergic cells concomitantly with L-DOPA delivery, aimed at replenishing the missing dopamine simultaneously with reversing oxidative stress/inflammatory damages. Treatment with SD and SDA having multifaceted activities combined into a single molecule have the potential to delay the onset of both motor fluctuations and dyskinesias and slow premature loss of nigrostriatal denervation of the dorsal putamen and nigral cells.

**Rotenone rat model of Parkinson's disease (PD); *In vivo* studies**

To assess the neuroprotective properties of SD and SDA in vivo, we used the systemic acute rotenone rat-model that reproducibly mimics many aspects of the pathology of human PD (32). In this model, the i.p injection of 3.0 mg/kg/day rotenone produces dopamine terminal loss in the dorsolateral striatum, a selective nigrostriatal degeneration, loss of tyrosine hydroxylase positive neurons without nonspecific lesions, as well as induction of α-syn and poly-ubiquitin aggregation (32), (38).

Since the rotenone-treated animals develop bradykinesia, postural instability, and/or rigidity, (32), this model serves as an excellent tool to test novel neuroprotective reagents that could provide insights into the pathogenesis of PD.

Motor coordination and balance were monitored in the rotenone/only, rotenone/SD and rotenone/SDA treated rats, using rotarod, beam walking, and rearing activity. Postural instability, and/or rigidity were developed by all rotenone/only-treated animals, and reversed in the rotenone/SD- or rotenone/SDA-treated rats. The significant rescue of impaired motor activities subsequent to intraperitoneal injection strongly suggests that both SD and SDA may well cross the BBB, reversing the rotenone-triggered toxicity of the DA neurons. The intraperitoneal injection of the thioredoxin mimetic peptide, TXM-CB3, was previously shown to reverse inflammatory consequences in the brain of diabetes Zucker rats (24). These results support a potential anti-inflammatory activity of SD, the newly designed member of the TXM-peptide family, and its ability to cross the BBB which might help managing PD motor-impairment.

**Anti-inflammatory and anti-apoptotic activity of SD and SDA; *In vitro* studies**

It is well established that the inflammatory/apoptotic pathway is activated by the apoptosis signal-regulating kinase1 (ASK1) (39). ASK1 forms a stable complex with reduced thioredoxin 1 (Trx1_red_). This ASK1/Trx1_red_ complex is unable to activate the MAPK apoptotic pathway. Upon oxidation of Trx1_red_ to Trx1_ox_, the complex dissociates enabling free ASK1 to activate the MAPK inflammatory/apoptotic pathway.

The anti-inflammatory/antioxidant properties of SD and SDA have been demonstrated in-vitro, monitoring the activation of MAPK pathway in the neuronal human neuroblastoma SH-SY5Y cells. Oxidative stress was induced in the cells by Auf, an organogold selective inhibitor of thioredoxin reductase, known to activate ASK1 by maintaining Trx1 in the oxidized form. Through the activation of the MAPK inflammatory/apoptotic pathway, Auf also inhibits the mitochondrial Trx2 causing the failure of mitochondrial function in the brain (40). Furthermore, Auf inhibits mitophagic flux, lowering mitochondrial membrane potential and ATP levels (35).

The ability of SD and SDA to reverse the Auf-oxidizing effects implies a dual mechanism of action. SD and SDA exert an indirect antioxidant effect by reducing Trx1_ox_ to Trx1_red_ with resultant activation of ASK1, and/or a direct effect of ROS scavenging and elevation of GSH levels. These effects establish the anti-apoptotic/anti-inflammatory activities of SD and SDA in neuronal cells, promoting potential clinical relevance in protecting nigrostriatal denervation of the dorsal putamen and slowing nigral cell loss.

**Combined therapies in a single molecule**

The in vivo studies suggest that SD or SDA, which have both NAC and levodopa moieties within them, appear to cross the BBB, thereby facilitating delivery of both components to the targeted cells. This is in contrast to NAC which is BBB-impermeable. Acting as a vehicle to bring NAC into the brain, SD and SDA might provide antioxidant protection to dopaminergic neurons, simultaneously with replenishing the missing dopamine in the nigral cells. The data suggest that the combined activities of SD and SDA into a single molecule, might have a significant advantage over NAC and levodopa administrated separately. In the absence of a clinically efficient neuroprotective treatment to slow PD progression, SD and SDA might have a putative disease-modifying effects by conferring antioxidant/anti-apoptotic activity within the DA neurons.

**Preventing α-syn aggregation**

Alpha-synuclein (α-syn) is abundantly expressed in neurons and is the major constituent of Lewy bodies, which are the hallmarks of neurodegenerative diseases called synucleinopathies, which like PD, results in multiple system atrophy, Lewy body dementia, Alzheimer’s disease, and frontotemporal dementia.

The prevalence of misfolded fibrillar aggregates of α-syn associated with Lewy bodies is consistent with the role of copper ions and oxidative stress, forming highly toxic α-syn oligomers and α-syn aggregation (41). Copper ions are elevated in the cerebrospinal fluid of PD patients and linked to α-syn oligomerization (42)*,* (43), (18), (44).

Since sulfhydryl groups are known to chelate copper ions, SD and SDA were examine for the effects on α-syn expression and aggregation.

Both SD and SDA showed a reduction in α-syn expression in SH-SY5Y cells exposed to a high dose of rotenone. Also, production of aggregated forms of α-syn was significantly lower in HEK-293 cells overexpressing α-syn in the presence of SDA. The precise mechanism by which SD or SDA prevent stress-induced α-syn expression in neuronal cells is not yet clear and requires further study.

The second experiment investigated a direct effect of SDA on α-syn oligomerization, using α-syn transiently transfected HEK293 cells, which express high levels of α-syn monomers and oligomers (45). Preincubation of the cells with SDA, resulted in a significantly lower level of α-syn dimers and trimers, but no change in the level of α-syn monomers. This experiment implies SDA interference in α-syn oligomerization, possibly by copper ions-chelating activity. Because α-syn transfected HEK293 cells do not represent the complexity and morphological features of α-syn inclusions or Lewy bodies in neuronal cells, further studies are required to establish the relevance lowering α-syn oligomerization to dopaminergic neurons.

**Conclusions**

The newly designed levodopa precursors, SD and SDA appear to relieve motor impairment in the rotenone rat model, and inhibit oxidative stress-induced activation of the inflammatory MAPK apoptotic-pathway in neuronal cells. Initial data also suggest that SDA reverses α-syn oligomerization.

To date, there is no clinically disease-modifying treatment that can halt PD progression. The failure to develop neuroprotective or disease-modifying strategies results mainly because treatment starts with the appearance of motor symptoms, long after a significant nigral cell loss. Nevertheless, the standard treatment of levodopa, which is almost entirely centered on dopamine replenishment performs rather efficiently during the initial 4-5 years of treatment, implying viability of the remaining dopaminergic neurons. Therefore replacing levodopa treatment with SD or SDA may possibly attenuate the loss of the remaining nigral cells, and slow PD progression. Future toxicology studies and subsequent clinical testing of SD or SDA alongside levodopa, could establish their potential clinical benefits and advantage in halting disease progression.

The combined redox activity and levodopa release with lowering α-syn aggregation might potentially slow-down nigral cell loss, protect nigrostriatal denervation of the dorsal putamen, and attenuate Lewy pathology. Hence, SD and SDA have the potential to become a disease-modifying treatment of PD or other LB-like pathologies.

**Methods**

**Materials**

Auranofin (Enzo Life Sciences, Shoham, Israel), triethylphosphine (2,3,4,6-tetra-O-acetyl-β-1-d-thiopyranosato-S) gold(I); Levodopa ethyl ester (LDEE) from TEVA Ltd Israel. SuperDopa (SD), SuperDopAmide (SDA), and thioredoxin mimetic peptide TXM-CB3, and AD4 (NAC-amide), were custom synthesized by Novetide, Ltd., Haifa, Israel. All other materials were purchased from Sigma, Jerusalem. Alpha-synuclein plasmid was a kind gift of Dr. R. Sharon (Hadassah Ein Kerem, Jerusalem).

**Cell culture and treatment**

Human neuroblastoma SH-SY5Y cells were cultured in DMEM/F12 HAM 1:1 medium supplemented with 10% fetal bovine serum (FBS) and penicillin–streptomycin, incubated at 37°C with 5% CO_2_, as previously reported (24). Human embryonic kidney HEK293 cells were cultured at 37°C, 5% CO_2_ in RPMI 1640 supplemented with: l-alanyl-l-glutamine (4.4mM); 10% fetal bovine serum; Penicillin (100U/ml) and Streptomycin (100μg/ml); HEPES pH 7.3 (10mM). Cells were plated at a density of 6.25X10^4^/cm^2^ and incubated for 24h, after which they were exposed to different treatments. Tissue culture serum and medium were from Biological Industries (Kibbutz Beit-Haemek, Israel).

***In vitro* Studies using SH-SY5Ycells**

The anti-inflammatory activity of SD and SDA was tested essentially as previously reported for TXM-CB3 (24). SH-SY5Y cells were incubated for 30 min with a 3μM auranofin (AuF), washed and incubated for 3.5h with or without SD, SDA, TXM-CB3, at the indicated concentrations. After washing with one ml of PBS the cells were lysed in 0.12 ml lysis buffer (150mM Tris, pH 6.8, 10% Glycerol, 0.6% SDS, Bromophenol Blue, supplemented with 7μl β-Mercaptoethanol/ml). Protein concentration of the lysates was determined by using Coomassie Brilliant-Blue staining. Cell lysates were heated to 100°C for 10 min prior to electrophoresis separation on SDS-PAGE gels.

**Western blot analysis and antibodies**

Western blot analysis were performed essentially as previously published (23). Twenty to thirty micrograms of protein samples were loaded on 10 or12% SDS–PAGE gels. The proteins were then transferred electrophoretically to nitrocellulose (Whatman, Germany). The blots were cut prior to hybridization with antibodies, and blocked by incubation for 1h at RT in TBS-T (25mM Tris–HCl pH 7.4, 0.9% NaCl and 0.02% Tween-20) with 4% Difco skim milk (BD, USA), and incubated over-night at 4°C with the primary antibody: pERK1/2 (Thr 202/Tyr204), mouse mAb; ERK2 (Santa Cruz, USA) rabbit Ab; p-SAPK/JNK (Thr183/Tyr185), rabbit mAb; SAPK/JNK, mouse mAb; p-p38^MAPK^ (Thr180/Tyr182), rabbit mAb; p38, rabbit Ab; GAPDH (Glyceraldehyde 3-phosphate dehydrogenase); α-syn, Abcam, Cambridge, U.K EPR20535; β Catenin, mouse mAb (1:10,000) BD Transduction Laboratories, USA) diluted in 5% BSA, 0.04% Azide in TBS-T. Proteins were detected with Anti-Mouse or Anti-Rabbit IgG-HRP linked antibody (1:10,000; Cell Signaling Tech., USA).

**Alpha-synuclein (α-syn**) **aggregation in transfected HEK293 cells**

HEK293 cells were plated on collagen (rat tail) )Roche Diagnostics, Mannheim, Germany) and incubated for 16hr in 24-well plates and allowed to reach 70–80% confluency in 1 ml of culture medium. The next day, the cells were washed twice with regular DMEM, incubated at 37ºC, for 5 hr in Opti MEM (Thermo Fisher Scientific, Waltham, MA) containing the transfection mixtures of 0.75μg plasmid cDNA encoding wt α-syn and polyethyleneimine (PEI) at a ratio of 1:3 (DNA to PEI) in 400µl Opti MEM after vortex for 10 sec, and incubated at RT for 15 min. After 5 hr the medium was replaced by regular DMEM containing 10% FBS solution. Cell media was replaced after overnight incubation, with DMEM containing 10% FBS solution with or w/o 500µM of SuperDopaAmide (SDA) and incubated for 48 hr. Then the cells were lysed with 0.12 ml ice-cold lysis buffer (150 mM Tris–HCl, pH 6.8, 10% glycerol, 0.6% SDS, bromophenol blue, supplemented with 7 μl β-mercaptoethanol, and heated overnight at 65°C. Proteins were separated on SDS-PAGE α-syn monomers, dimers, and trimers were detected after blotting to nitrocellulose with the corresponding α-syn antibodies.

**Induction of α**-**synuclein by rotenone in SH-SY5Y cells**

SH-SY5Y cells were treated with increasing concentration of SD or SDA for 60 min. Then the cells were washed and incubated with 5µM rotenone for 16 hrs. The level of α-synuclein in cell lysates was determined after protein separation on 12% SDS-PAGE using α -syn antibodies. The values calculated by densitometry shown as averages (±SEM) of two independent experiments normalized with housekeeping level of β-catenin; Student’s t-test (two populations) was performed for rotenone treated cells. *p value < 0.05; **p value < 0.01.

**Animals**

***Ethics*** A commercial company Science in Action, Nes-Ziona, Israel, uses its ethical permission to do animal studies as an outsourcing service (#C148210).

All animals were treated according to the National Institute of Health (NIH) guidelines for the care and use of laboratory animals. Animal ethics committee accredits the company, and licensed veterinarians conducted the experiments. Rat were sacrificed by an overdose of CO_2_ and decapitated.

**Rotenone treatment**

The experiments were performed by “Science in Action” Rehovot, according to Cannon et al., (32). Rotenone (Sigma, St Lewis, MO, USA) was dissolved (3mg/ml) in DMSO (10%w/in sunflower oil pH7.4). During the treatment period (10 days) rotenone was stored at 6^o^C. Twenty male Sprague Dawley rats 7-8 weeks (315 gr; Envigo, Rehovot) were divided into 4 experimental groups (each, n=6) and 2 naïve rats. Rotenone was administrated at 3.0 mg/kg intraperitoneally (IP) once a day in the morning (days 1-9). SD (33mg/kg) or SDA (33mg/kg) were administered once a day (IP) in the afternoon (days 1-9). SD at 33mg/kg and SDA at 33mg/kg were administrated intraperitoneally once a in the afternoon (days 1-9).

| Route of administration | Treatment | Animals  (n) | Group  (#) |
| --- | --- | --- | --- |
| ip | Rotenone (3mg/kg) | n = 6 (#1-6) | (1) Control |
| ip  ip | Rotenone (3mg/kg)  +SDA (33.3mg/kg) | n = 6 (#7-12) | (2) SDA |
| ip  ip | Rotenone (3mg/kg)  +SD (33.3mg/kg) | n = 6 (#13-18) | (3) SD |
| - | None | n = 2 (#19-20) | (4) Naive |

**Animal behavior**

Animals body weight was determined before initiation of treatment (day 0), on days 4, 7, 10 during the experiment and on termination day 11. On day 11 the animals were sacrificed.

Rearing behavior, rotarod, and beam walk tests were performed before initiation of treatment (day 0) and on days 4, 7, and 10 of the experiment

**Rotarod behavior test**

Motor coordination and balance was evaluated on the rotarod, which was set to accelerate from 4 to 40 rpm in 300 sec. Animals were placed at separate lanes on the rotarod with initial rotation set on 4 rpm.

**Rat rearing behavior test**

Animals were placed in a clear glass cylinder (40cm high x 20cm diameter) and number of rears in 2 min was monitored. Rear was considered when animals raised their front legs above the shoulder and made a contact with the wall of the cylinder with their forelimb.

**Rat beam-walk**

Animals were trained for 2 days to traverse the length of the beam. On the day of the test animals were gently placed on the 1m long narrow aluminum beam facing one of the ends and allowed to walk along the beam, monitoring the time taken to reach the end of the beam.

**Statistical Analysis**

All values were presented as mean ± standard deviation (SD) or standard error of mean (SEM), and differences were considered to be statistically significant at the *P < 0.05 level ^31^. Statistical analysis was performed using StatsDirect statistical software (Cheshire, UK). Differences among means were analyzed using 1‐way ANOVA followed by comparison to the control rotenone-only‐treated rats group (Dunnet's test). Within groups, comparison to the baseline was performed by 2‐way ANOVA, and nonparametric data were analyzed with Kruskal–Wallis ANOVA or Friedman ANOVA, respectively.

**List of abbreviations**

Auf, auranofin; α-syn, alpha synuclein; ERK1/2, extracellular signal-regulated kinases; HEK293, human embryonic kidney; JNK, c-Jun N-terminal kinases; L-DOPA, levodopa; MAPK, mitogen activated protein kinase; NAC, N-acetylcysteine; PD, Parkinson’s disease; p38^MAPK^, p38 mitogen activated protein kinase; SD, SuperDopa; SDA, SuperDopAmide; TrxR, thioredoxin reductase; Trx1, Thioredoxin; TXM, Thioredoxin mimetic;

**Declarations**

**Acknowledgments**

The authors wish to thank the“ Kamin Fund of “The Israel Innovation Authority”.

**Funding Statement**

This research was funded by the “Kamin” Fund of “The Israel Innovation Authority” to DA.

“A novel anti apoptotic slow release L-dopa precursors” #65118. The funder had no role in study design, data collection and analysis, decision to publish, or preparation of the manuscript.

**Data Availability**

All data generated or analyzed during this study are included in this published article.

**Authors contribution**

T.W carried out the majority of the experiments and performed data analysis; D.A designed and wrote the paper

**Ethics approval**

The Medical Ethical Committee of the Ministry of Health Israel gave the study “Evaluation of the effect of SD and SDA in a rat model of Parkinson's Disease“ Approval number # IL-18-3-82.

The *in vivo* studies were performed by *Science in Action* Ltd Ness-Ziona, Israel.

The study was carried out in compliance with ARRIVE guidelines.

**Conflict of Interests**

The authors declare no competing financial interests

**References**

1. Antonini A, Moro E, Godeiro C, Reichmann H. Medical and surgical management of advanced Parkinson's disease. Mov Disord. 2018;33(6):900-8.

2. Chou KL, Stacy M, Simuni T, Miyasaki J, Oertel WH, Sethi K, et al. The spectrum of "off" in Parkinson's disease: What have we learned over 40 years? Parkinsonism Relat Disord. 2018;51:9-16.

3. Armstrong MJ, Okun MS. Diagnosis and Treatment of Parkinson Disease: A Review. JAMA. 2020;323(6):548-60.

4. Rincon M, Flavell RA, Davis RA. The JNK and P38 MAP kinase signaling pathways in T cell-mediated immune responses. Free Radic Biol Med. 2000;28(9):1328-37.

5. Offen D, Gilgun-Sherki Y, Barhum Y, Benhar M, Grinberg L, Reich R, et al. A low molecular weight copper chelator crosses the blood-brain barrier and attenuates experimental autoimmune encephalomyelitis. J Neurochem. 2004;89(5):1241-51.

6. Bahat-Stroomza M, Gilgun-Sherki Y, Offen D, Panet H, Saada A, Krool-Galron N, et al. A novel thiol antioxidant that crosses the blood brain barrier protects dopaminergic neurons in experimental models of Parkinson's disease. Eur J Neurosci. 2005;21(3):637-46.

7. Bartov O, Sultana R, Butterfield DA, Atlas D. Low molecular weight thiol amides attenuate MAPK activity and protect primary neurons from Abeta(1-42) toxicity. Brain Res. 2006;1069(1):198-206.

8. Langston JW, Ballard PA, Jr. Parkinson's disease in a chemist working with 1-methyl-4-phenyl-1,2,5,6-tetrahydropyridine. N Engl J Med. 1983;309(5):310.

9. Dauer W, Przedborski S. Parkinson's disease: mechanisms and models. Neuron. 2003;39(6):889-909.

10. Ryan SD, Dolatabadi N, Chan SF, Zhang X, Akhtar MW, Parker J, et al. Isogenic human iPSC Parkinson's model shows nitrosative stress-induced dysfunction in MEF2-PGC1alpha transcription. Cell. 2013;155(6):1351-64.

11. Giasson BI, Duda JE, Murray IV, Chen Q, Souza JM, Hurtig HI, et al. Oxidative damage linked to neurodegeneration by selective alpha-synuclein nitration in synucleinopathy lesions. Science. 2000;290(5493):985-9.

12. Schildknecht S, Gerding HR, Karreman C, Drescher M, Lashuel HA, Outeiro TF, et al. Oxidative and nitrative alpha-synuclein modifications and proteostatic stress: implications for disease mechanisms and interventions in synucleinopathies. J Neurochem. 2013;125(4):491-511.

13. Chavarria C, Souza JM. Oxidation and nitration of alpha-synuclein and their implications in neurodegenerative diseases. Arch Biochem Biophys. 2013;533(1-2):25-32.

14. Devi L, Raghavendran V, Prabhu BM, Avadhani NG, Anandatheerthavarada HK. Mitochondrial import and accumulation of alpha-synuclein impair complex I in human dopaminergic neuronal cultures and Parkinson disease brain. J Biol Chem. 2008;283(14):9089-100.

15. Spillantini MG, Crowther RA, Jakes R, Hasegawa M, Goedert M. alpha-Synuclein in filamentous inclusions of Lewy bodies from Parkinson's disease and dementia with lewy bodies. Proc Natl Acad Sci U S A. 1998;95(11):6469-73.

16. Masliah E, Iwai A, Mallory M, Ueda K, Saitoh T. Altered presynaptic protein NACP is associated with plaque formation and neurodegeneration in Alzheimer's disease. Am J Pathol. 1996;148(1):201-10.

17. Duda JE, Lee VM, Trojanowski JQ. Neuropathology of synuclein aggregates. J Neurosci Res. 2000;61(2):121-7.

18. Braak H, Del Tredici K, Rub U, de Vos RA, Jansen Steur EN, Braak E. Staging of brain pathology related to sporadic Parkinson's disease. Neurobiol Aging. 2003;24(2):197-211.

19. Dodel R, Csoti I, Ebersbach G, Fuchs G, Hahne M, Kuhn W, et al. Lewy body dementia and Parkinson's disease with dementia. J Neurol. 2008;255 Suppl 5:39-47.

20. Hayashita-Kinoh H, Yamada M, Yokota T, Mizuno Y, Mochizuki H. Down-regulation of alpha-synuclein expression can rescue dopaminergic cells from cell death in the substantia nigra of Parkinson's disease rat model. Biochem Biophys Res Commun. 2006;341(4):1088-95.

21. Bachnoff N, Trus M, Atlas D. Alleviation of oxidative stress by potent and selective thioredoxin-mimetic peptides. Free Radic Biol Med. 2011;50(10):1355-67.

22. Kim SR, Lee KS, Park SJ, Min KH, Lee MH, Lee KA, et al. A novel dithiol amide CB3 attenuates allergic airway disease through negative regulation of p38 mitogen-activated protein kinase. Am J Respir Crit Care Med. 2011;183(8):1015-24.

23. Cohen-Kutner M, Khomsky L, Trus M, Aisner Y, Niv MY, Benhar M, et al. Thioredoxin-mimetic peptides (TXM) reverse auranofin induced apoptosis and restore insulin secretion in insulinoma cells. Biochem Pharmacol. 2013;85(7):977-90.

24. Cohen-Kutner M, Khomsky L, Trus M, Ben-Yehuda H, Lenhard JM, Liang Y, et al. Thioredoxin-mimetic peptide CB3 lowers MAPKinase activity in the Zucker rat brain. Redox Biol. 2014;2:447-56.

25. Lejnev K, Khomsky L, Bokvist K, Mistriel-Zerbib S, Naveh T, Farb TB, et al. Thioredoxin-mimetic peptides (TXM) inhibit inflammatory pathways associated with high-glucose and oxidative stress. Free Radic Biol Med. 2016;99:557-71.

26. Baratz-Goldstein R, Deselms H, Heim LR, Khomski L, Hoffer BJ, Atlas D, et al. Thioredoxin-Mimetic-Peptides Protect Cognitive Function after Mild Traumatic Brain Injury (mTBI). PLoS One. 2016;11(6):e0157064.

27. Canesi F, Mateo V, Couchie D, Karabina S, Negre-Salvayre A, Rouis M, et al. A thioredoxin-mimetic peptide exerts potent anti-inflammatory, antioxidant, and atheroprotective effects in ApoE2.Ki mice fed high fat diet. Cardiovasc Res. 2019;115(2):292-301.

28. Hemling P, Zibrova D, Strutz J, Sohrabi Y, Desoye G, Schulten H, et al. Hyperglycemia-induced endothelial dysfunction is alleviated by thioredoxin mimetic peptides through the restoration of VEGFR-2-induced responses and improved cell survival. Int J Cardiol. 2019.

29. Kronenfeld G, Engelman R, Weisman-Shomer P, Atlas D, Benhar M. Thioredoxin-mimetic peptides as catalysts of S-denitrosylation and anti-nitrosative stress agents. Free Radic Biol Med. 2015;79:138-46.

30. Atlas D. Emerging therapeutic opportunities of novel thiol-amides, NAC-amide (AD4/NACA) and thioredoxin mimetics (TXM-Peptides) for neurodegenerative-related disorders. Free Radic Biol Med. 2021;176:120-41.

31. Atlas D. DopAmide: Novel, Water-Soluble, Slow-Release l-dihydroxyphenylalanine (l-DOPA) Precursor Moderates l-DOPA Conversion to Dopamine and Generates a Sustained Level of Dopamine at Dopaminergic Neurons. CNS Neurosci Ther. 2016;22(6):461-7.

32. Cannon JR, Tapias V, Na HM, Honick AS, Drolet RE, Greenamyre JT. A highly reproducible rotenone model of Parkinson's disease. Neurobiol Dis. 2009;34(2):279-90.

33. Xicoy H, Wieringa B, Martens GJ. The SH-SY5Y cell line in Parkinson's disease research: a systematic review. Mol Neurodegener. 2017;12(1):10.

34. Bell M, Zempel H. SH-SY5Y-derived neurons: a human neuronal model system for investigating TAU sorting and neuronal subtype-specific TAU vulnerability. Rev Neurosci. 2021.

35. Yumnamcha T, Devi TS, Singh LP. Auranofin Mediates Mitochondrial Dysregulation and Inflammatory Cell Death in Human Retinal Pigment Epithelial Cells: Implications of Retinal Neurodegenerative Diseases. Front Neurosci. 2019;13:1065.

36. Djaldetti R, Atlas D, Melamed E. Effect of subcutaneous administration of levodopa ethyl ester, a soluble prodrug of levodopa, on dopamine metabolism in rodent striatum: implication for treatment of Parkinson's disease. Clin Neuropharmacol. 1996;19(1):65-71.

37. Olanow CW. Levodopa: effect on cell death and the natural history of Parkinson's disease. Mov Disord. 2015;30(1):37-44.

38. Zhu C, Vourc'h P, Fernagut PO, Fleming SM, Lacan S, Dicarlo CD, et al. Variable effects of chronic subcutaneous administration of rotenone on striatal histology. J Comp Neurol. 2004;478(4):418-26.

39. Saitoh M, Nishitoh H, Fujii M, Takeda K, Tobiume K, Sawada Y, et al. Mammalian thioredoxin is a direct inhibitor of apoptosis signal-regulating kinase (ASK) 1. EMBO J. 1998;17(9):2596-606.

40. Drechsel DA, Patel M. Respiration-dependent H2O2 removal in brain mitochondria via the thioredoxin/peroxiredoxin system. J Biol Chem. 2010;285(36):27850-8.

41. Lashuel HA, Overk CR, Oueslati A, Masliah E. The many faces of alpha-synuclein: from structure and toxicity to therapeutic target. Nat Rev Neurosci. 2013;14(1):38-48.

42. Pall HS, Williams AC, Blake DR, Lunec J, Gutteridge JM, Hall M, et al. Raised cerebrospinal-fluid copper concentration in Parkinson's disease. Lancet. 1987;2(8553):238-41.

43. Okita Y, Rcom-H'cheo-Gauthier AN, Goulding M, Chung RS, Faller P, Pountney DL. Metallothionein, Copper and Alpha-Synuclein in Alpha-Synucleinopathies. Front Neurosci. 2017;11:114.

44. Falcone E, Ahmed IMM, Oliveri V, Bellia F, Vileno B, El Khoury Y, et al. Acrolein and Copper as Competitive Effectors of alpha-Synuclein. Chemistry. 2020;26(8):1871-9.

45. Zarbiv Y, Simhi-Haham D, Israeli E, Elhadi SA, Grigoletto J, Sharon R. Lysine residues at the first and second KTKEGV repeats mediate alpha-Synuclein binding to membrane phospholipids. Neurobiol Dis. 2014;70:90-8.

**Legends to figures**

**Figure 1**

**The structure and putative proteolysis sites of SuperDopa (SD) and SuperDopaAmide (SDA)**

*Upper* putative proteolysis sited for SuperDopa (SD) and *Lower*, putative proteolysis sited for SuperDopaAmide (SDA. The red arrows indicate peptide bond, potential cleavage sites at the peptides bonds.

**Figure 2**

**The effect of rotenone, rotenone + SD, and rotenone + SDA on body mass**

(**A**) Daily intraperitoneal rotenone (3mg/kg) for 9 days elicits moderate change in body mass from day 0 to day10 in rats (300 gr; n=6) (**B**) Quantification of body mass taken at day 0, 4, 7, and 10 after rotenone injection.

**Figure 3**

**SD and SDA rescue motor impairment in rotenone-treated rats**

Rats were injected with rotenone alone (3mg/kg; 10 days), or with either SD (33.3mg/kg daily for 9 days) or SDA (33.3mg/kg daily; 9 days). Motor activity was examined at days 4, 7, and 10 by (**A**) Rotarod assay monitored motor coordination. Apparatus was set to accelerate from 4 to 40 rpm in 300s, and animals placed in separate lanes riding time (**B**) The Cylinder test which evaluated locomotor asymmetry by placing the rats in a clear glass cylinder (40 cm high and 20 cm diameter) and number of rears were counted during 5 min and (**C**) The walk bean assay which measured successful walk rats walk across an elevated 1m long aluminum beam, counting the number of foot slips from the beam as a measure of locomotor co-ordination and grip-strength.

**Figure 4**

**SD and SDA reverses the auranofin-induced phosphorylation of JNK in** **human neuroblastoma SH-SH5Y cells**

**(A, B**) SH-SY5Y cells were incubated with 3μM auranofin (Auf) for 30 min, washed, and treated with or without increasing concentrations of SD in 4 independent experiments.

(**C,D**) SH-SY5Y cells were incubated with 3μM auranofin (Auf) for 30 min, washed, and treated with or without increasing concentrations of SDA in 3 independent experiments, as indicated. Cell lysates proteins in equal amounts were separated on 10% SDS-PAGE, and analyzed by immunoblotting with the corresponding antibodies. The blots were cut prior to hybridization with antibodies shown in **Fig. S8**. The ratios of phosphorylated JNK to unphosphorylated JNK, or β-catenin were calculated. The values shown are averages (±SEM) based on four independent experiments, normalized to the phosphorylation state of cells treated with Auf after 3.5 h and plotted with a linear regression program. Student's *t*-test (two populations) was performed for AuF treated cells. **P* value <0.05; ***P* value <0.01; ****P* value < 0.005.

**Figure 5**

**SD and SDA reverse Auf-induced p38^MAPK^ phosphorylation in human neuroblastoma SH-SY5Y cells**

(**A, B**) SH-SY5Y cells were incubated with 3μM Auf for 30 min, washed, and treated with or without increasing concentrations of SD. (**C, D**) SH-SY5Y cells were incubated with 3μM Auf for 30 min, washed, and treated with or without increasing concentrations of SDA for 3.5h. Proteins in equal amounts of cell lysates were separated on 10% SDS-PAGE, and analyzed by immunoblotting with the corresponding antibodies. The blots were cut prior to hybridization with antibodies shown in **Fig. S9**. Phosphorylation of p38^MAPK^ was quantified by immunoblot densitometry. The ratios of phosphorylated p38^MAPK^ to the housekeeping β-catenin were calculated based on three independent experiments. The values shown are averages (±SEM) normalized to the phosphorylation state of cells treated with Auf after 3.5h and plotted with a linear regression program. Student's *t*-test (two populations) was performed for Auf treated cells. **P* value <0.05; ***P* value <0.01; ****P* value < 0.005.

**Figure 6**

**SD, SDA but not LDEE, reverse Auf-induced JNK and p38^MAPK^ phosphorylation**

A concentration dependent reduction in Auf-induced phosphorylation of (**A**) JNK and (**B**) p38^MAPK^ by SD, SDA, and LDEE (see legend and blots in **Fig. S11**). The ratios of phospho-JNK/JNK1 and phospho-p38^MAPK^/β-catenin were calculated as percentage of control based on two independent experiments ($\pm$SEM) for each of the compounds.

**Figure 7**

**SD and SDA lower rotenone induced α-synuclein level in SH-SY5Y cells**

SH-SY5Y cells were treated with (**A**) SD or (**B**) SDA at the concentrations as indicated, for 60 min. Then the cells were washed and incubated with 5µM rotenone for 16 hrs. Equal amounts of proteins of cell lysates were loaded and separated on 12% SDS-PAGE. The level of α-syn in the cell lysates was determined using α-synuclein antibodies. The blots were cut prior to hybridization with the corresponding antibodies, shown in **Fig. S12**. The values calculated by densitometry shown (*right*) are averages (±SEM) of two independent experiments normalized with housekeeping level of β-catenin; Student’s t-test (two populations) was performed for rotenone treated cells. *p value < 0.05; **p value < 0.01.

**Figure 8**

**SDA lower accumulation of dimer and trimer forms of α-synuclein in HEK293 over-expressing α-syn**

HEK293 cells were transfected with wt α-syn, and 24 hr later washed, and treated with or without 500μM SDA and incubated for additional 24 h. The cells were lysed 48 hr after transfection and heated to 65^o^C overnight. (**A**) Equal amounts of protein were loaded and separated on 12% SDS-PAGE. α-syn was determined by immunoblotting using the corresponding α-syn antibody. Control represents non-transfected cells (**B**) Quantification of α-syn monomers normalized to β-catenin (**C**) Quantification of α-syn dimers and trimers normalized to β-catenin. The amount of each band was quantitated by densitometry and plotted with a linear regression program. The values shown are average (±SEM) of an experiment consisting of triplicates normalized to β-catenin.
