# Supplementary material for: Novel anti-apoptotic L-DOPA precursors SuperDopa and SuperDopamide as potential neuroprotective agents for halting/delaying progression of Parkinson’s disease
Source: Cell Death Dis. 2022 Mar 11;13(3):227. doi: 10.1038/s41419-022-04667-2 (PMC8917195; doi:10.1038/s41419-022-04667-2)

**The Novel** **Anti-apoptotic Levodopa Precursors SuperDopa and SuperDopamide as potential candidates for halting/delaying** **the progression of Parkinson’s disease**

**Tom Wiesen and Daphne Atlas***

daphne.atlas@mail.huji.ac.il

**Supplementary Materials**

**Figure S1**

**High Performance Liquid Chromatography (HPLC) of SD**

**
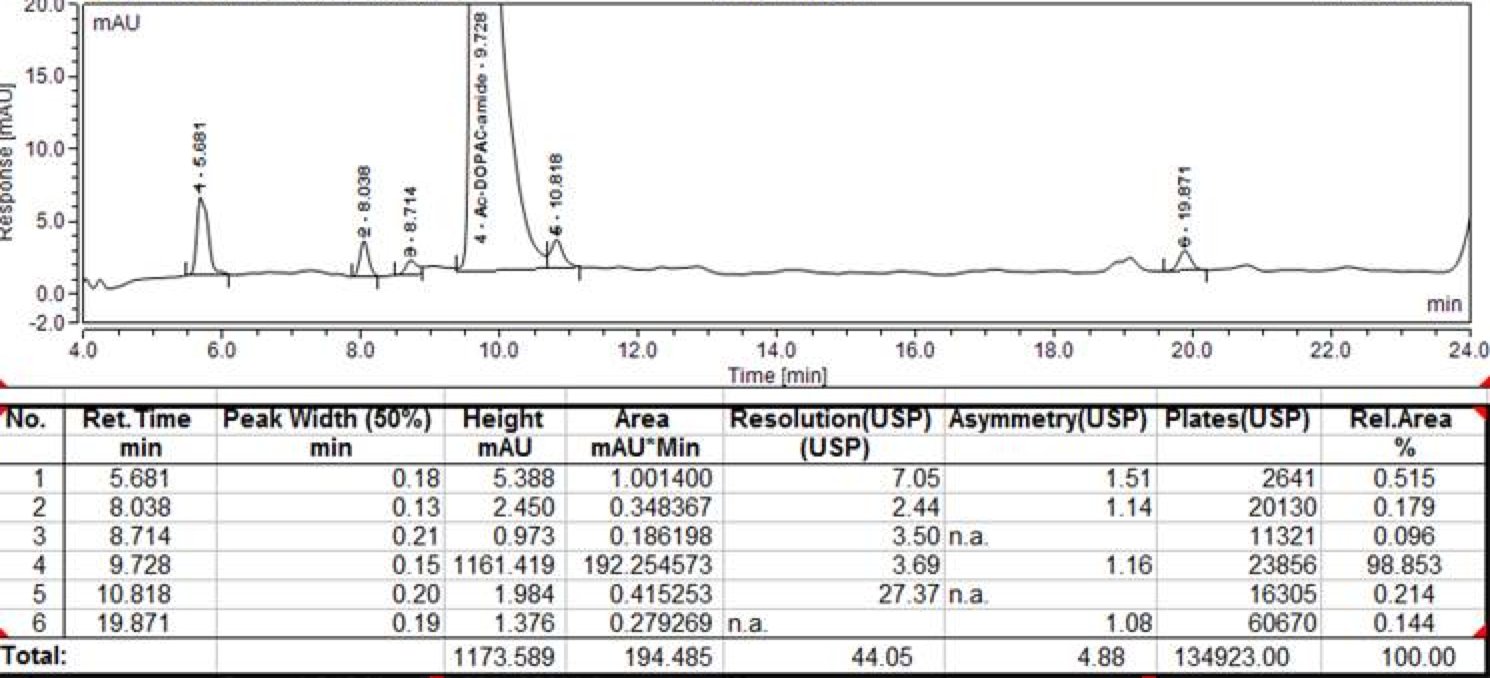
**

**Figure S2**

**Mass spectra of SD**


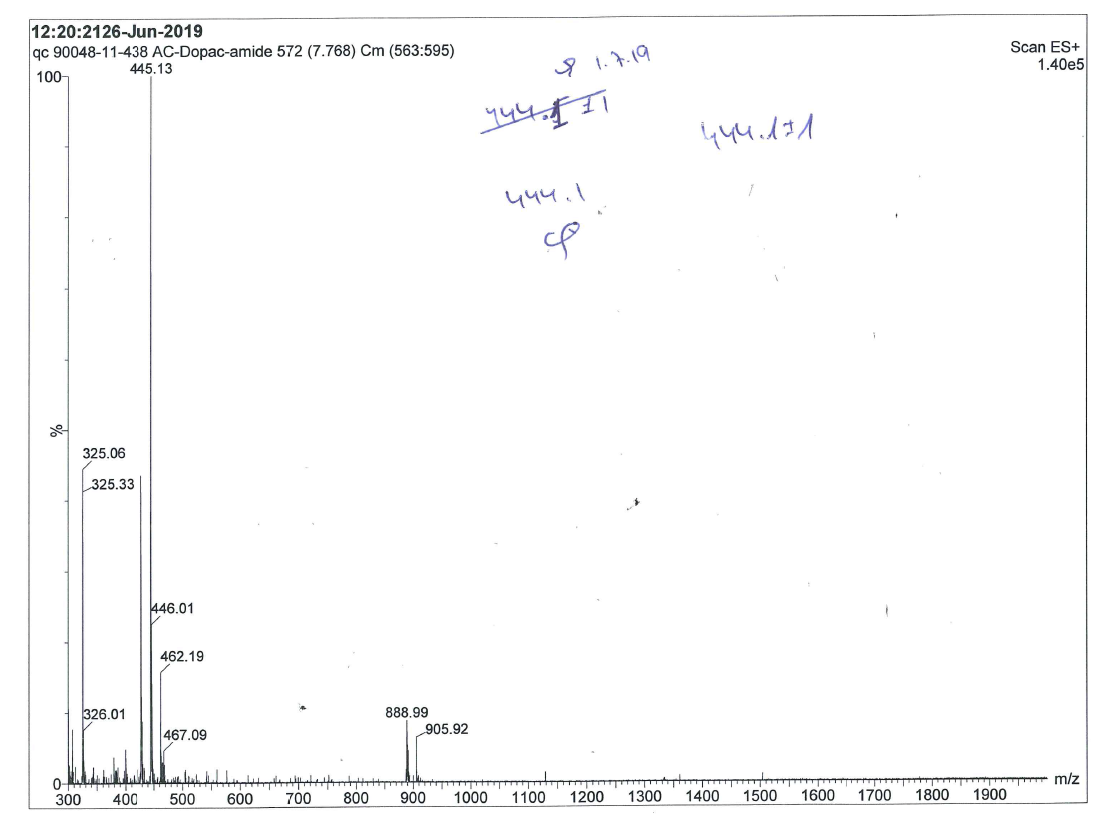


**Figure S3**

**High Performance Liquid Chromatography (HPLC) of SDA**

**
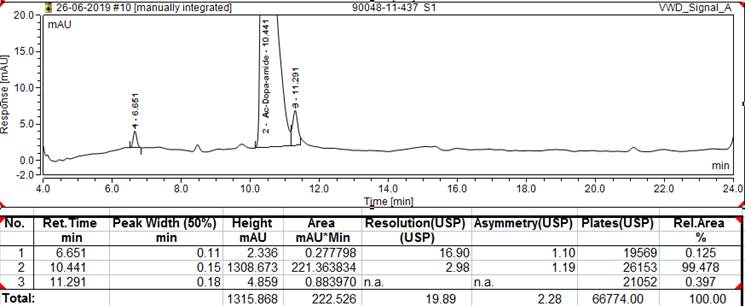
**

**Figure S4**

**Mass spectra of SDA**


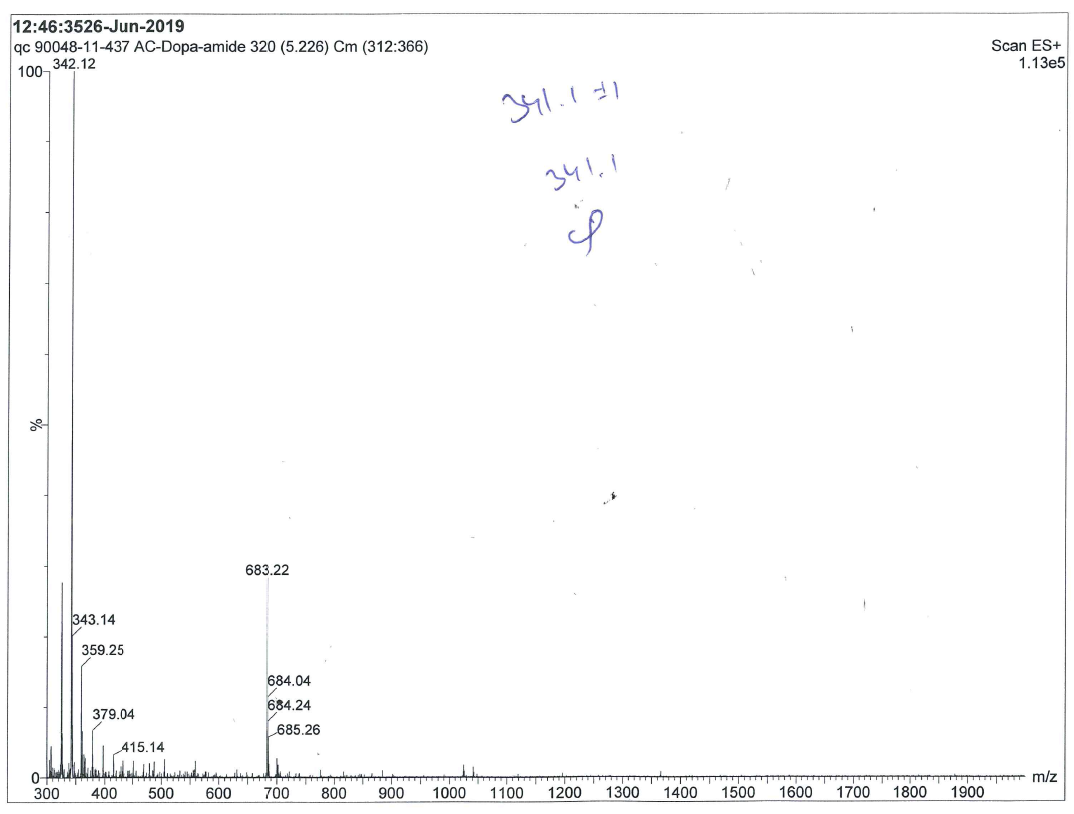


**Figure S5**

**FIugre S4**

**Mass spectra of SDA**

**Figure S5**

**Rotenone and Auranofin lower the Mitochondrial membrane potential (MMP) in PC12 cell**

Mitochondrial Membrane Potential (MMP) was tested using the JC-1 mitochondrial membrane potential assay kit (Abcam, Cambridge, U.K.). PC12 cells (2.0 × 10^4^ cells/well) were seeded onto clear bottom 96-well plates and incubated at 37 °C overnight. Cells were washed and stained with 2 μM JC-1 in serum-free media for 10 min at 37 °C and 5% CO_2_. After staining cells were washed, and treated with 10µM FCCP, 3µM auranofin, or 0.1µM rotenone, and incubated for 4hr at 37 °C and 5% CO_2_. The fluorescent signal was recorded using the Synergy H1 BioTek plate reader. Values are averages (+SEM) of 3 independent experiments; Student’s t-test was performed; *P value < 0.01.


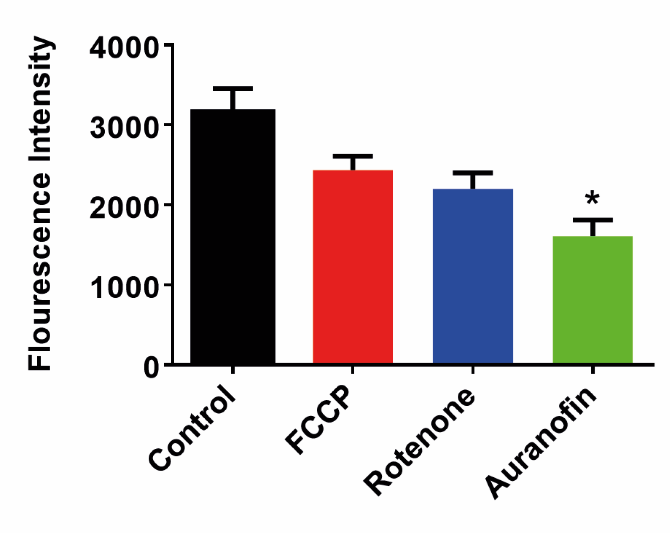


**Figure S6**

**The effect of SD and SDA and TXM-CB3 on the morphology of cells treated with AuF**

SH-SY5Y cells were incubated with 3µM Auf washed and incubated for additional 3.5 h with or without 200µM SDA, 200µM TXM-CB3, or 200µM SD,

Auf-induced morphological changes in SH-SY5Y were visualized in phase-contrast microscopy (magnification x40) Scale bars = 50µm

**
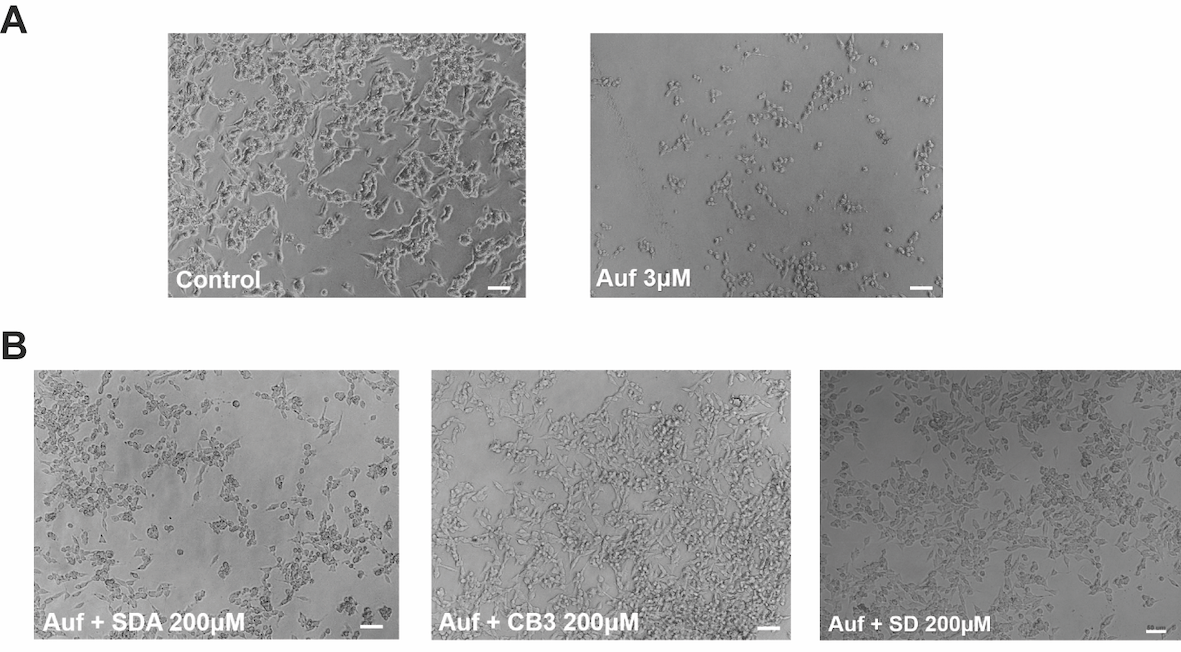
**

**Figure S7**

**The effect of rotenone on SH-SY5Y cell morphology**

SH-SY5Y cells were incubated with or without 5µM rotenone for 4 h.

No significant morphological changes were visualized in phase-contrast microscopy (magnification x40) Scale bars = 50µm


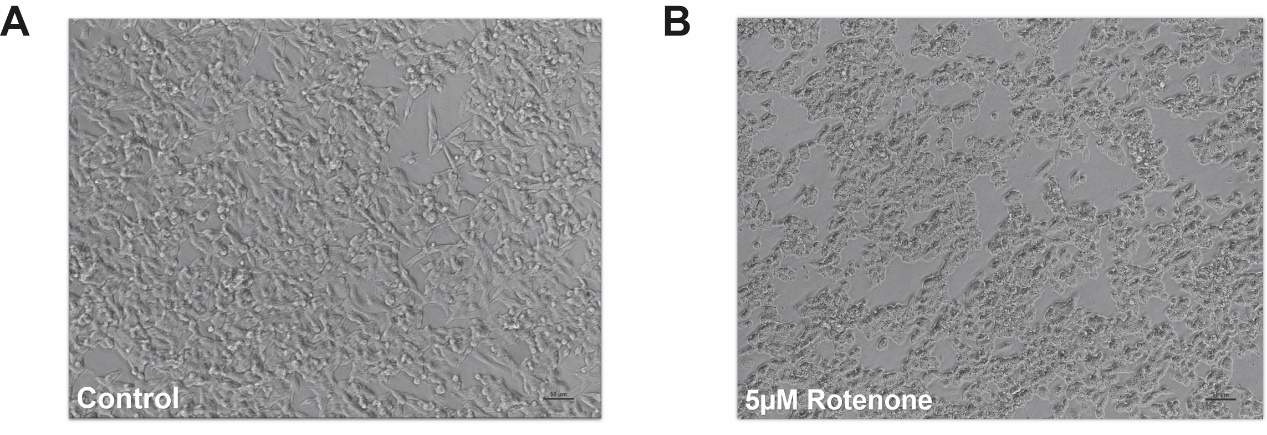


**Fig. S8**

**SD and SDA reverses the auranofin-induced phosphorylation of JNK in** **human neuroblastoma SH-SH5Y cells**

The blots were cut prior to hybridization with the corresponding antibodies as shown; see Fig. 4.

**
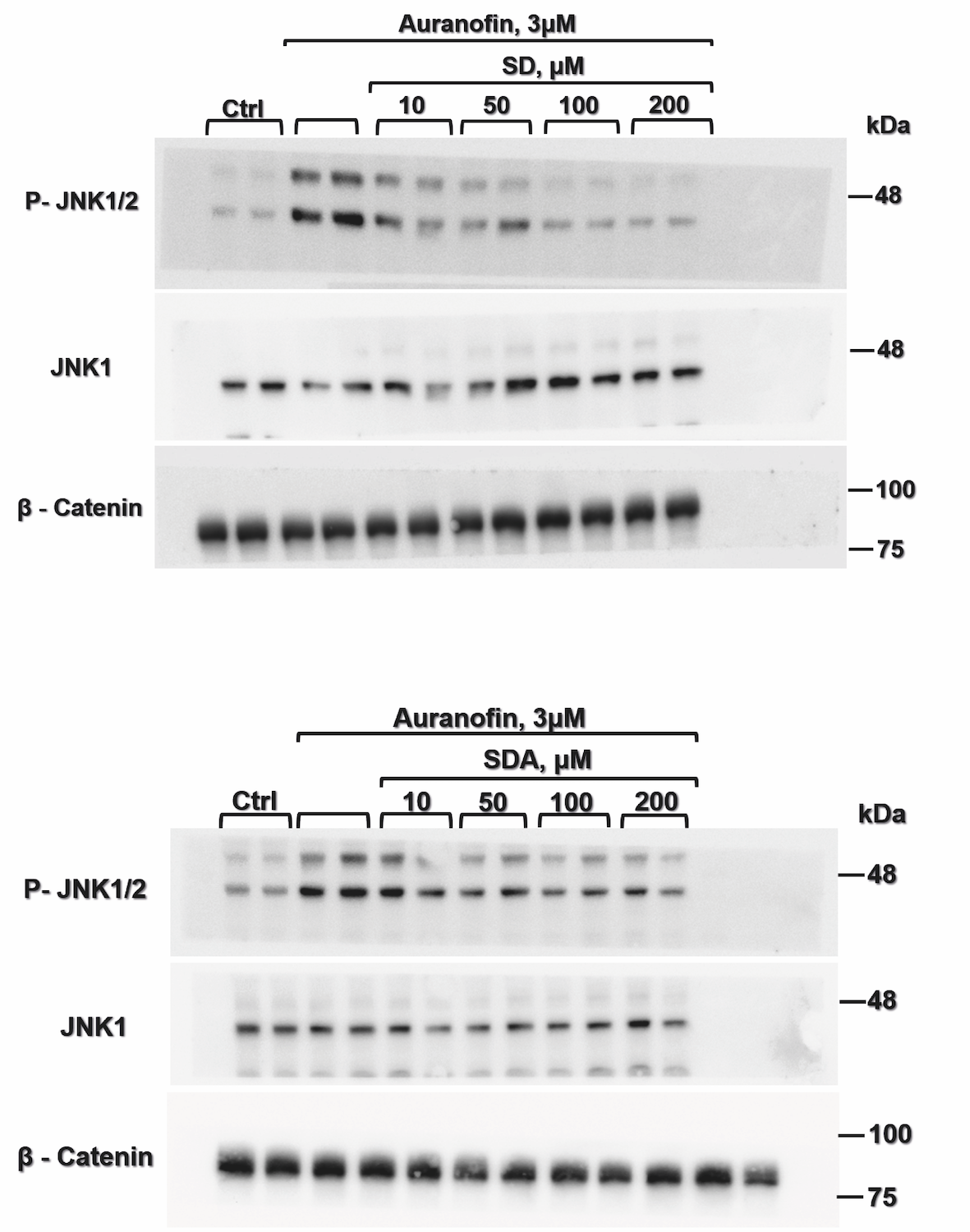
**

**Fig. S9**

**SD and SDA reverse Auf-induced p38^MAPK^ phosphorylation in human neuroblastoma SH-SY5Y cells**

The blots were cut prior to hybridization with the corresponding antibodies as shown; see Fig. 5.


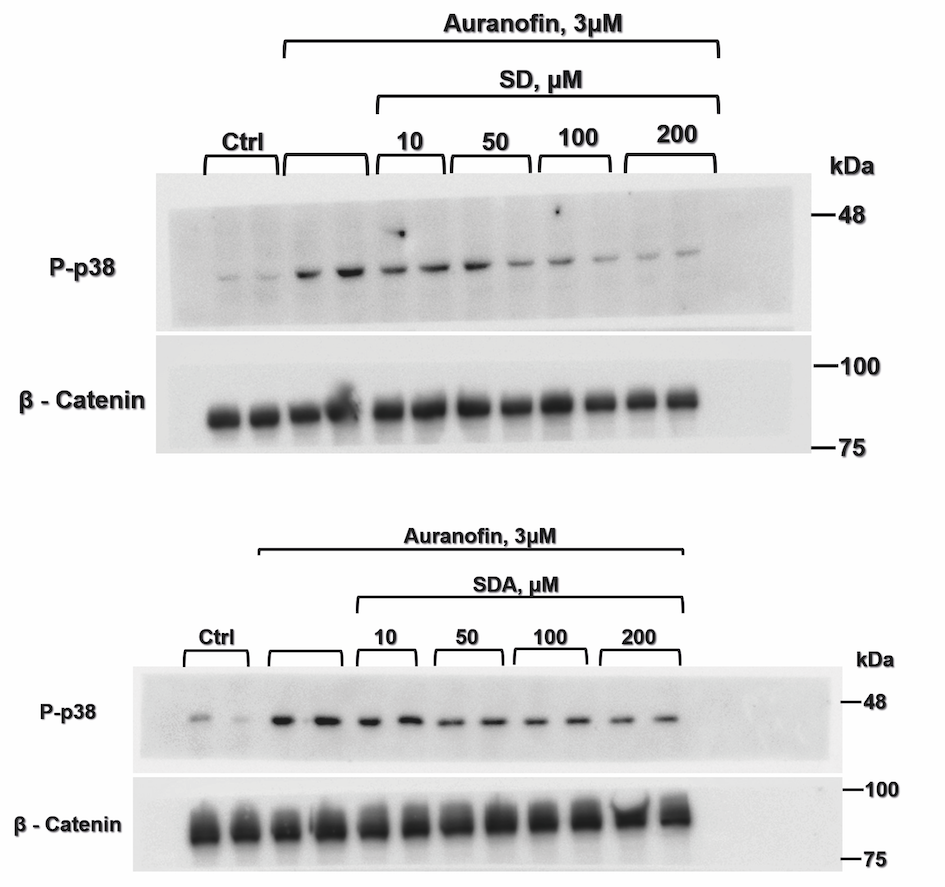


**Figure S10**

**The effect of SD on Auf-induced ERK1/2 phosphorylation in human neuroblastoma SH-SY5Y cells**

SH-SY5Y cells were incubated with 3μM Auf for 30 min, washed, and treated with or without increasing concentrations of SD for 3.5 h. Proteins in equal amounts of cell lysates were separated on 10% SDS-PAGE, and analyzed by immunoblotting with the corresponding antibodies. The blots were cut prior to hybridization with the corresponding antibodies. Phosphorylation of ERK1/2 was quantified by immunoblot densitometry. The ratios of phosphorylated ERK1/2 to the housekeeping β-catenin was calculated based on three independent experiments. The values shown are averages (±SEM) normalized to the phosphorylation state of cells treated with Auf after 3.5h and plotted with a linear regression program. Student's *t*-test (two populations) was performed for Auf treated cells.

No significant reversal in Auf-induced phosphorylation was observed


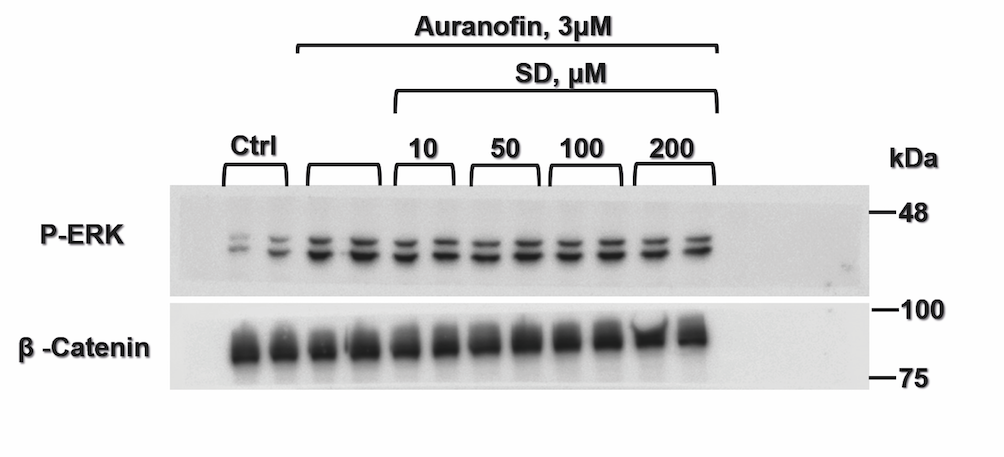


**
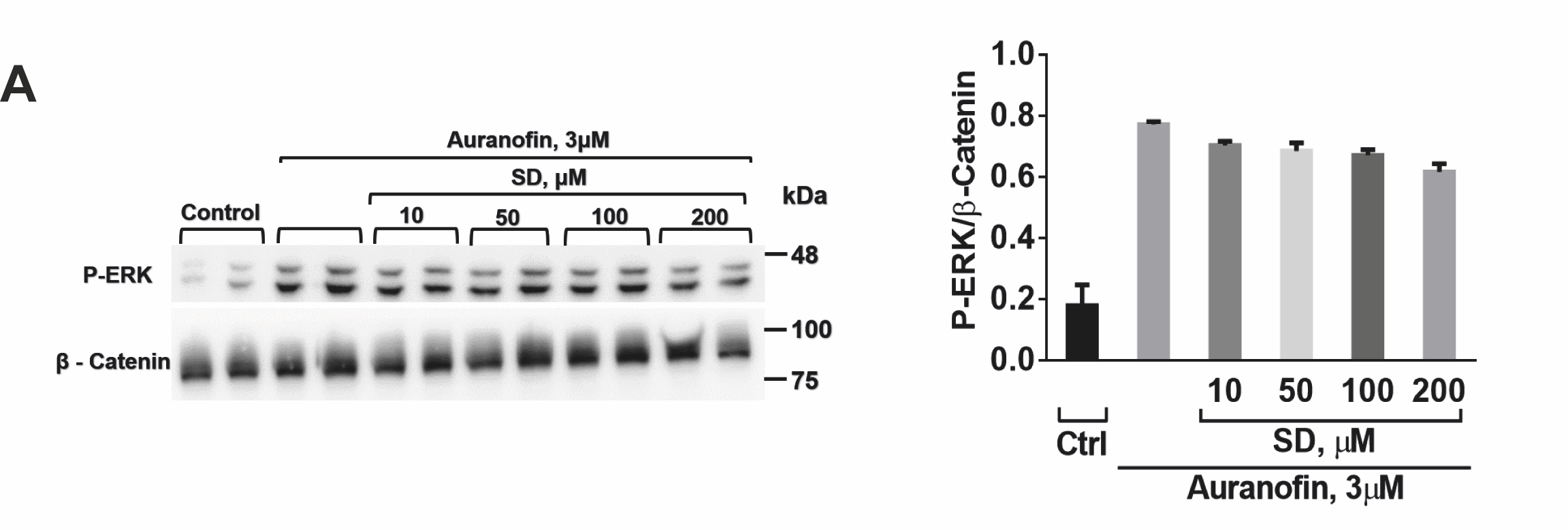
**

**Figure S11**

**Levodopa ethyl ester (LDEE) does not lower auranofin-induced p38^MAPK^ and JNK phosphorylation**

SH-SY5Y cells were incubated with 5μM Auf for 30 min, washed, and treated with or without increasing concentrations of levodopa ethyl ester (LDEE) (**A**) Phosphorylation of JNK and (**B**) Phosphorylation of p38^MAPK^ was determined after protein separation on 10% SDS-PAGE. The blots were cut prior to hybridization and analysis was performed by immunoblotting with the corresponding anti-phospho JNK and anti-phospho p38^MAPK^ antibodies (**see lower panel**). The ratios of phosphorylated JNK or p38^MAPK^ to JNK1 or the housekeeping β-catenin were calculated based on three independent experiments. The values shown are averages (±SEM) of three independent experiments normalized to the phosphorylation state of cells treated with Auf after 3.5h. Student's *t*-test (two populations) was performed for Auf-treated cells. **P* value <0.05; ***P* value <0.01; ****P* value < 0.005.


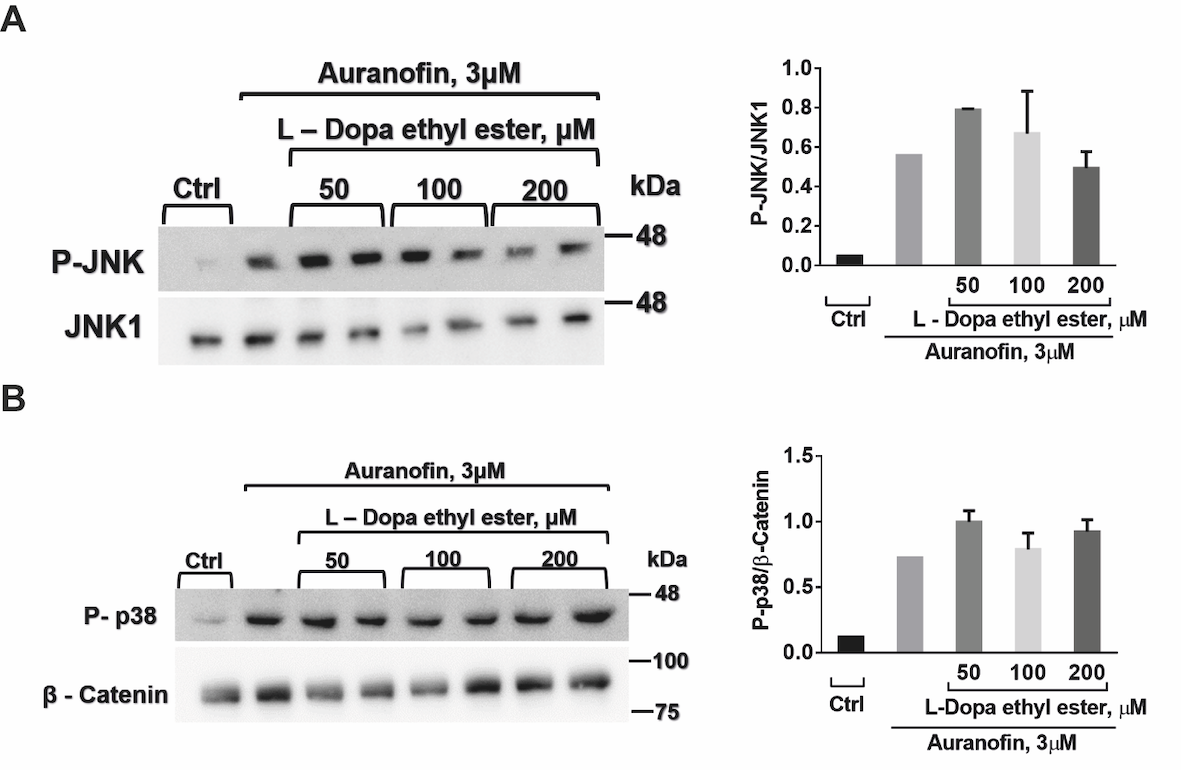


Original blots see below

**
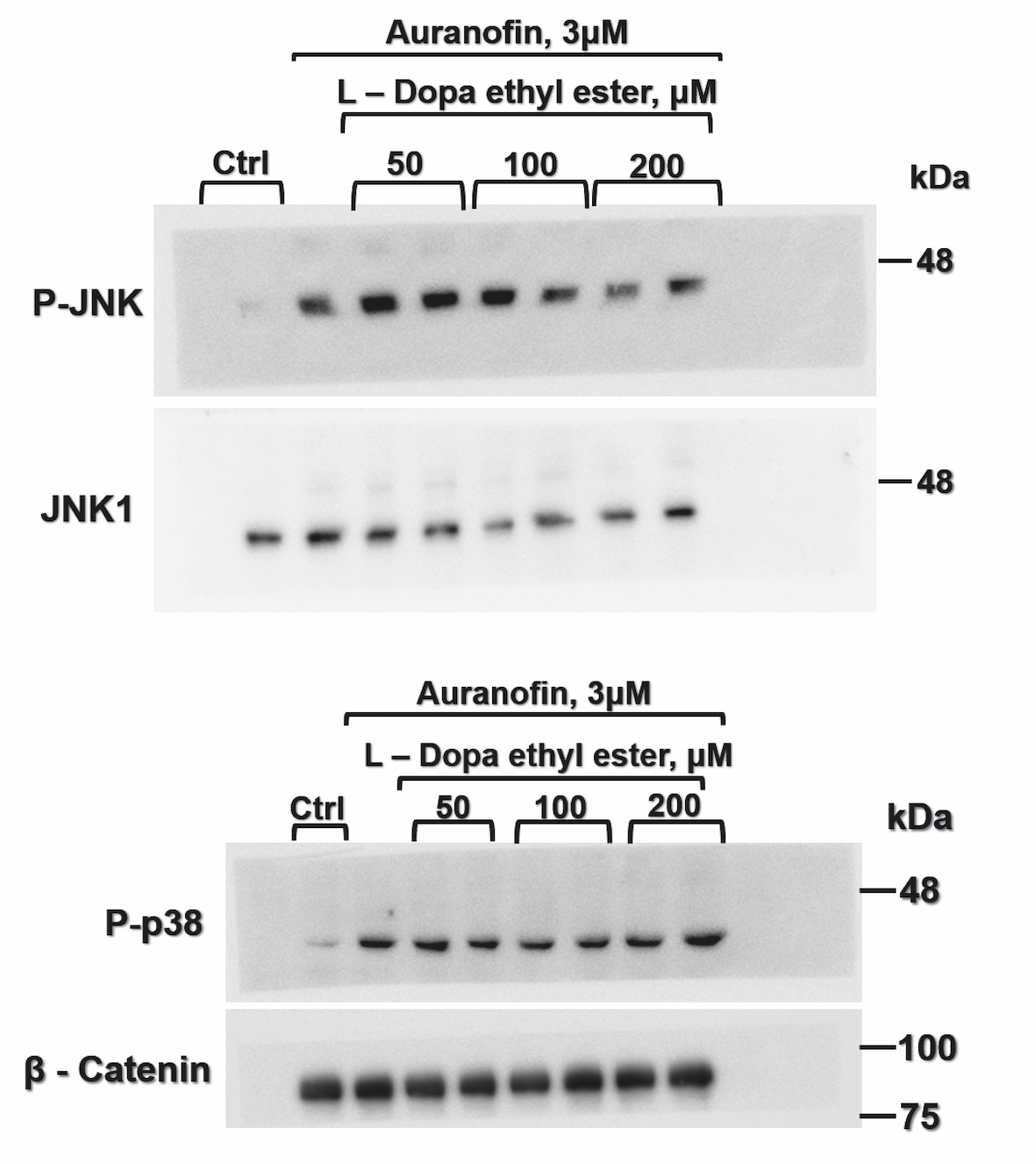
**

**Figure S12**

**SD and SDA lower rotenone induced α-synuclein level in SH-SY5Y cells**

The blots were cut prior to hybridization with the corresponding antibodies as shown; see Fig.7

**
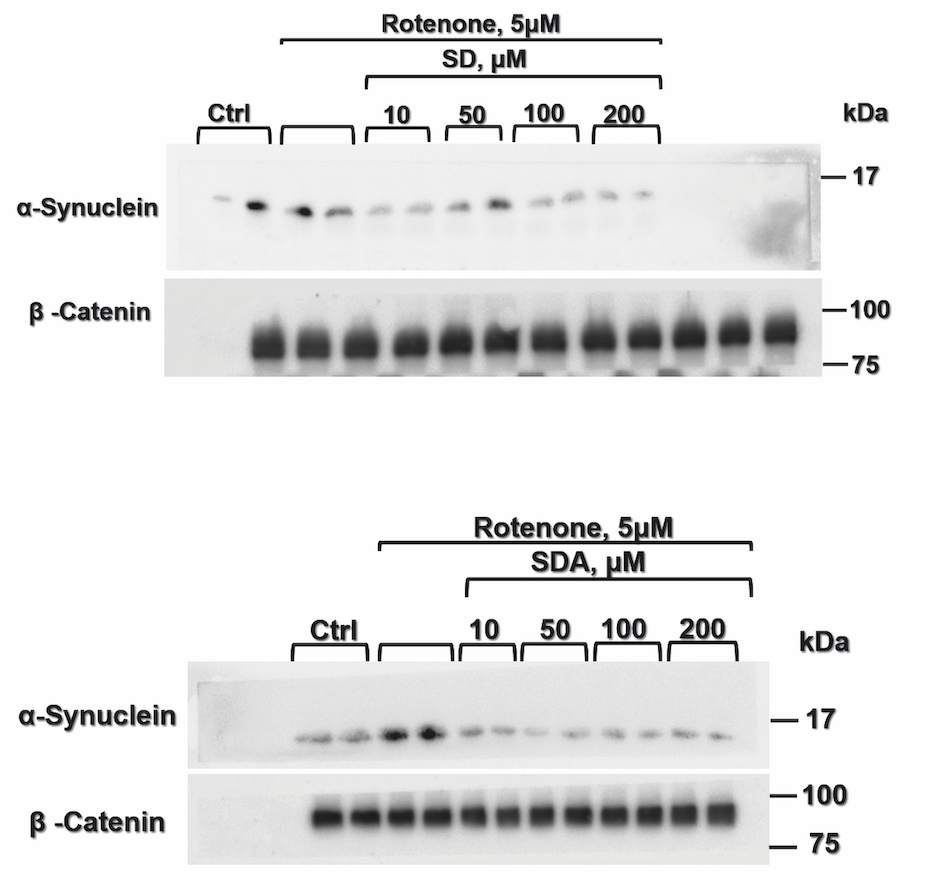
**

**Fig. S13**

**SDA lower accumulation of dimer and trimer forms of α-synuclein in HEK293 over-expressing α-synuclein**

Full blots of Fig. 8


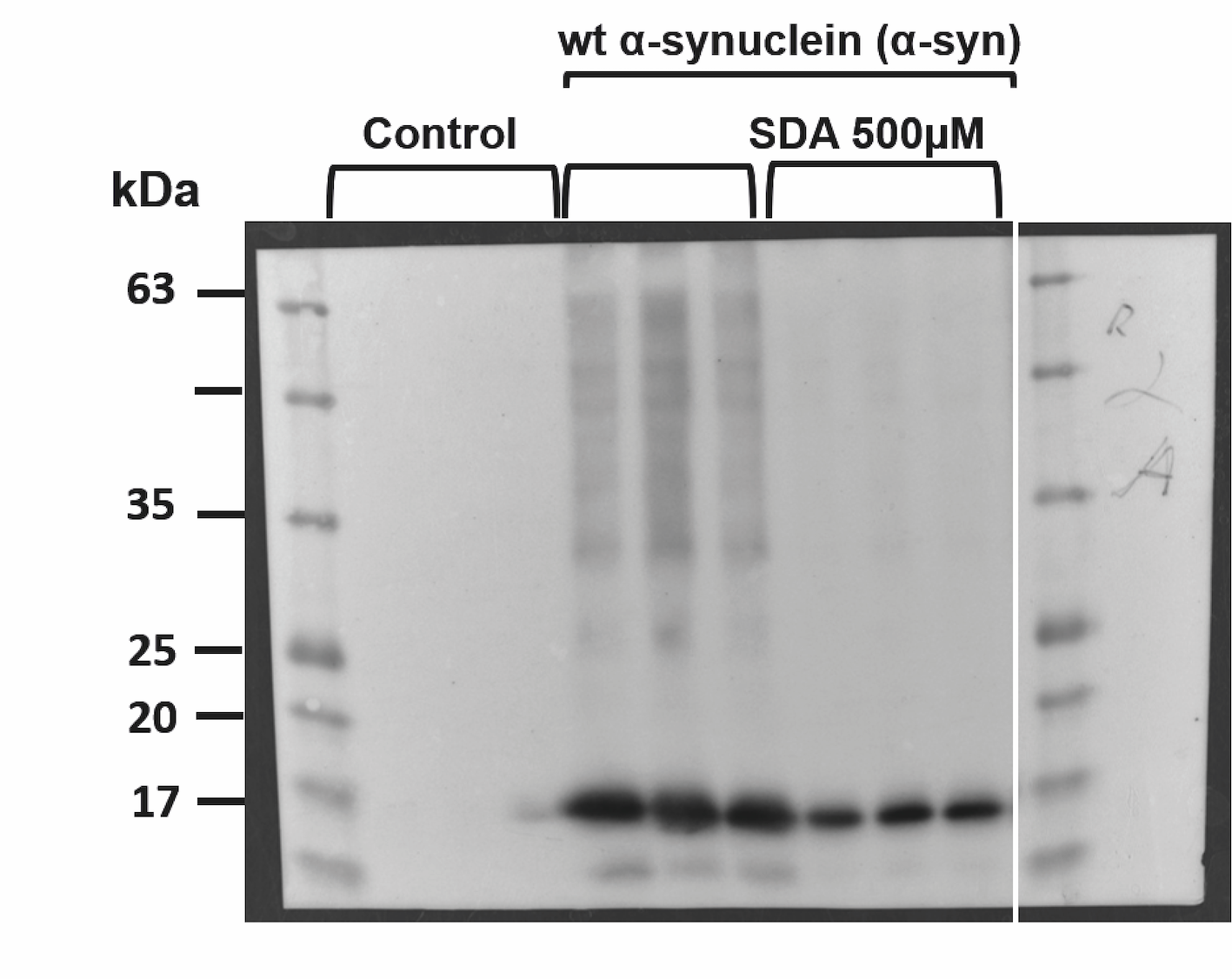


**β Catenin**


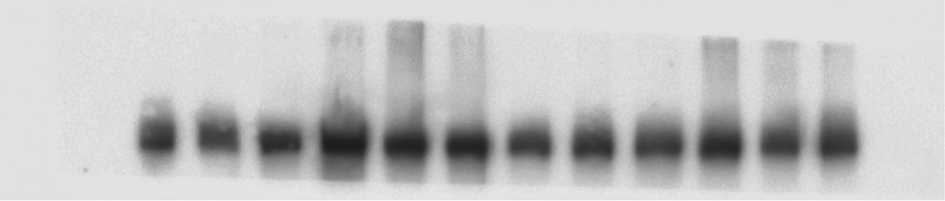

Supplement: Supplementary file 2 — Supplemental Material [file 41419_2022_4667_MOESM2_ESM.docx]
